# Directional Liquid Transport Enabled pH‐Responsive Hierarchical Composite for Enhanced Wound Healing

**DOI:** 10.1002/adhm.202505497

**Published:** 2026-02-03

**Authors:** Baolin Wang, Li‐Fang Zhu, Yuna Lang, Siyi Zhang, Fei Chen, Ming‐Wei Chang

**Affiliations:** ^1^ State Key Laboratory of Reliability and Intelligence of Electrical Equipment Hebei University of Technology Tianjin China; ^2^ College of Tourism and Leisure Management Fujian Business University Fuzhou Fujian China; ^3^ Tianjin Key Laboratory of Bio‐electromagnetic and Neural engineering Hebei University of Technology Tianjin China; ^4^ Hebei Key Laboratory of Bioelectromagnetics and Neuroengineering School of Health Sciences and Biomedical Engineering Hebei University of Technology Tianjin China; ^5^ Nanotechnology and Integrated Bioengineering Centre University of Ulster Belfast UK

**Keywords:** anti‐inflammatory, dual‐drug delivery, hierarchy, liquid diode flow, pH‐responsive

## Abstract

Persistent inflammation and infection within a macerated microenvironment critically hinder skin wound healing. Here, we report an engineered to regulate liquid transport and promote wound repair. The composite consists of a hydrophobic top layer, a hydrophilic gel‐forming middle layer, and two drug‐loaded fibrous layers with tunable hydrophobicity. This gradient architecture from hydrophobic to hydrophilic layers integrates directional liquid transport, efficient water absorption, breathability, and mechanical robustness. The diode‐like liquid transport behavior enables pH‐responsive, dual‐drug release, providing synergistic anti‐inflammatory and antibacterial effects. Consequently, this design minimizes maceration while maintaining a moist, bioactive environment favorable for tissue regeneration. Both in vitro and in vivo studies confirm the composite's pronounced antioxidant and hemostatic activities, along with its ability to markedly reduce infection and inflammation, thereby accelerating wound closure and promoting new tissue formation. This work presents a multifunctional therapeutic platform and highlights the significant clinical potential of this hierarchical composite for advanced wound management.

## Graphical abstract

A hierarchical composite with a gradient architecture transitioning from hydrophobic to hydrophilic layers integrates diode‐like liquid transport, efficient water absorption, breathability, and mechanical robustness. This device enables a multifunctional therapeutic platform with pH‐responsive dual‐drug release, providing synergistic anti‐inflammatory and antibacterial effects while minimizing maceration and maintaining a moist, bioactive environment conducive to advanced wound management and tissue regeneration.

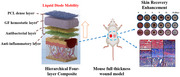

## 1 Introduction

The skin has long been recognized to play essential roles in sensation, homeostatic regulation, and appearance, serving as a protective barrier that shields internal organs from environmental threats [1, 2]. The treatment of skin wounds often involves costly and prolonged therapies due to the high risk of inflammation and infection, which require a series of coordinated physiological processes within local tissues [3, 4]. During the early stages of healing, bacterial invasion can easily occur, leading to persistent inflammation and impairing key repair processes such as re‐epithelialization, angiogenesis, and extracellular matrix (ECM) synthesis [5, 6]. Moreover, several detrimental factors including wound size, depth, and a persistently moist environment, can promote the accumulation of reactive oxygen species (ROS), disrupting the delicate balance between oxidative and antioxidant activity in the body and potentially resulting in cytotoxicity and tissue damage [7, 8].

Recent advances in wound healing based on the use of antimicrobial peptides, bacteriophages, biofilms, and bioactive materials, have demonstrated some promising functionality. However, their integration to effectively regulate wound exudate and prevent maceration within the wound microenvironment has introduced challenges [9, 10, 11, 12]. Furthermore, most current wound treatments rely on the sustained release of antibiotics, exacerbating the global challenge of antibiotic resistance. Early research on artificial skin was grounded in tissue engineering, with materials primarily serving as temporary wound coverings to support dermal repair [13, 14]. More recent studies have emphasized the importance of replicating the microstructural and biochemical characteristics of native skin to provide bionic microenvironmental cues that enhance wound healing [10, 15]. Despite notable advances in material design, a major challenge persists in achieving a synergistic replication of the complex biological architecture and multifunctional properties of natural skin, which intrinsically integrates defense and repair mechanisms through coordinated antimicrobial, anti‐inflammatory, and stimuli‐responsive functionalities [16, 17].

Controllable liquid transport through the passive, directional movement of fluids across asymmetric material systems has garnered considerable interest owing to its ubiquity in natural systems and wide‐ranging technological relevance [18]. Such materials, often referred to as liquid diodes, enable spontaneous, energy‐free liquid flow or penetration in a single direction, arising from gradients in interfacial structure and surface energy that drive liquids toward regions of stronger adhesion and away from weaker ones via forces acting along the fluid–solid contact line [19]. Recent studies have established that both compositional and topographical heterogeneities critically influence wetting behavior and flow dynamics, with engineered structures through topological features offering promising pathways to enhance the performance of liquid‐diode systems [20, 21]. Governed primarily by surface energy gradients, these systems rely on chemical heterogeneity to promote droplet motion toward more wettable regions; however, efficient directional transport further requires precise control of hierarchical micro/nanostructures and surface chemistry to mitigate contact‐line pinning and hysteresis, which often impede flow mobility [22]. Drawing inspiration from such natural mechanisms, the development of liquid‐transport materials capable of operating effectively in moist or macerated environments presents a highly promising avenue for advanced wound healing applications.

To achieve continuous, directional liquid transport coupled with pH‐responsive drug release for enhanced wound recovery, we present a hierarchically structured, gel‐form‐based composite that integrates liquid‐diode mobility, anti‐inflammatory, antibacterial, and stimuli‐responsive functionalities. The hydrophobic top polycaprolactone (TPCL) barrier and the hemostatic gel‐form (GF) layers are firmly bonded via electrostatic adsorption, mimicking the epidermis with excellent biocompatibility and mechanical robustness [23]. To synergistically regulate inflammation and antimicrobial activity through pH‐triggered, directionally dependent drug release, the bioactive compound curcumin (Cur) was encapsulated in Eudragit E100 (E100) within the skin‐contact layer, enabling anti‐inflammatory activity under mildly acidic conditions (pH ≈ 5). Cur suppresses key inflammatory pathways by inhibiting nuclear factor‐kappa B (NF‐ κB) activation, while also exhibiting strong antioxidant capacity, minimal cytotoxicity, and the ability to promote collagen synthesis [24, 25]. When exudate permeates the secondary layer composed of Eudragit S100 (S100) fibers loaded with rifampicin (Rif), the antibiotic is released under alkaline conditions (pH > 7), providing broad‐spectrum antibacterial efficacy against diverse mycobacterial and bacterial infections [26, 27].

This seamless integration of directional liquid transport enables dynamic regulation of wound healing during inflammation. Under acidic conditions arising from lactic acid accumulation due to anaerobic glycolysis in inflamed cells [28], Cur release is activated. Conversely, in infected wounds where the pH elevates to 7.3–9.8 [29], Rif release is triggered. The hierarchical architecture, characterized by its large surface area, interconnected porosity, and graded hydrophobicity from outer to inner layers, promotes unidirectional fluid transport through the porous matrix, exhibiting liquid‐diode behavior that modulates inflammatory responses within the macerated wound microenvironment. This multifunctional composite, endowed with integrated liquid‐diode mobility, was systematically investigated through both in vitro and in vivo studies, demonstrating significant potential for clinical wound management. Furthermore, its facile fabrication and practical applicability highlight its promise for scalable industrial and commercial translation.

## 2 Results and Discussions

### 2.1 Morphology and Size of the Hierarchical Composite

**SCHEME 1:**
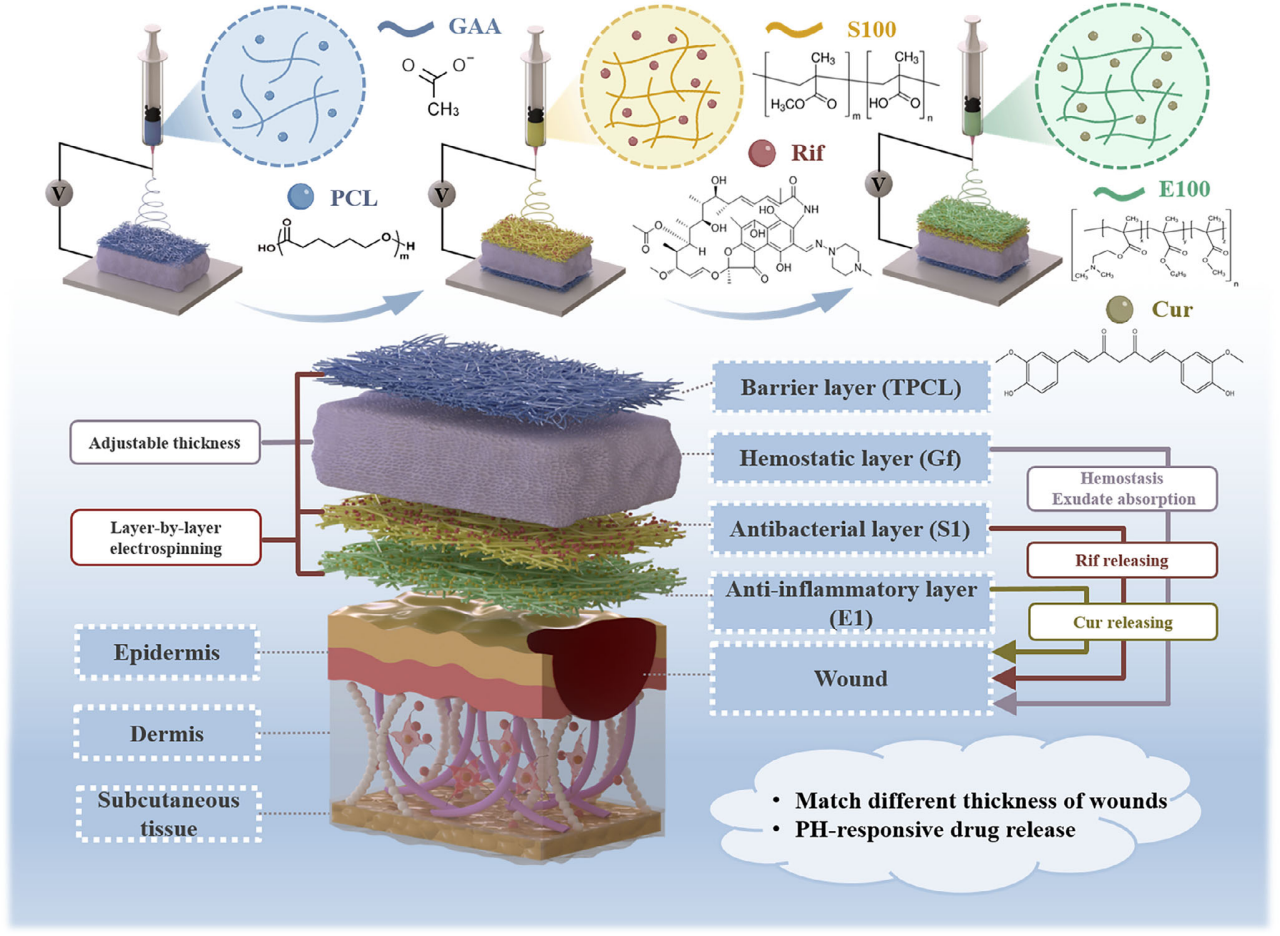
Schematic illustration of the preparation and functional mechanism of the hierarchical composite. The hierarchical porous layers exhibit directional flow from the skin‐contact layer to the hemostatic layer, triggering antibacterial and anti‐inflammatory effects through a synergistic pH‐responsive drug delivery mechanism, while maintaining a moist and nutrient‐rich environment in the macerated wound microenvironment to accelerate healing.

The hierarchical composite was fabricated following the stepwise procedure illustrated in Scheme 1. The top PCL barrier layer, denoted as TPCL, is a hydrophobic dense membrane prepared by electrospinning. Beneath this barrier layer lies a Gel‐foam (Gf) layer, which exhibits biocompatible porosity and high permeability. In this design, the TPCL and Gf layers are integrated via electrostatic adsorption to ensure both biocompatibility and mechanical integrity. A rifampicin‐loaded S100 electrospun membrane (denoted as S1) was fabricated as the antimicrobial layer using an S100/Rif solution. Subsequently, an anti‐inflammatory layer, designated as E1, was constructed at the bottom using E100/curcumin (Cur) nanofiber membranes.

**FIGURE 1:**
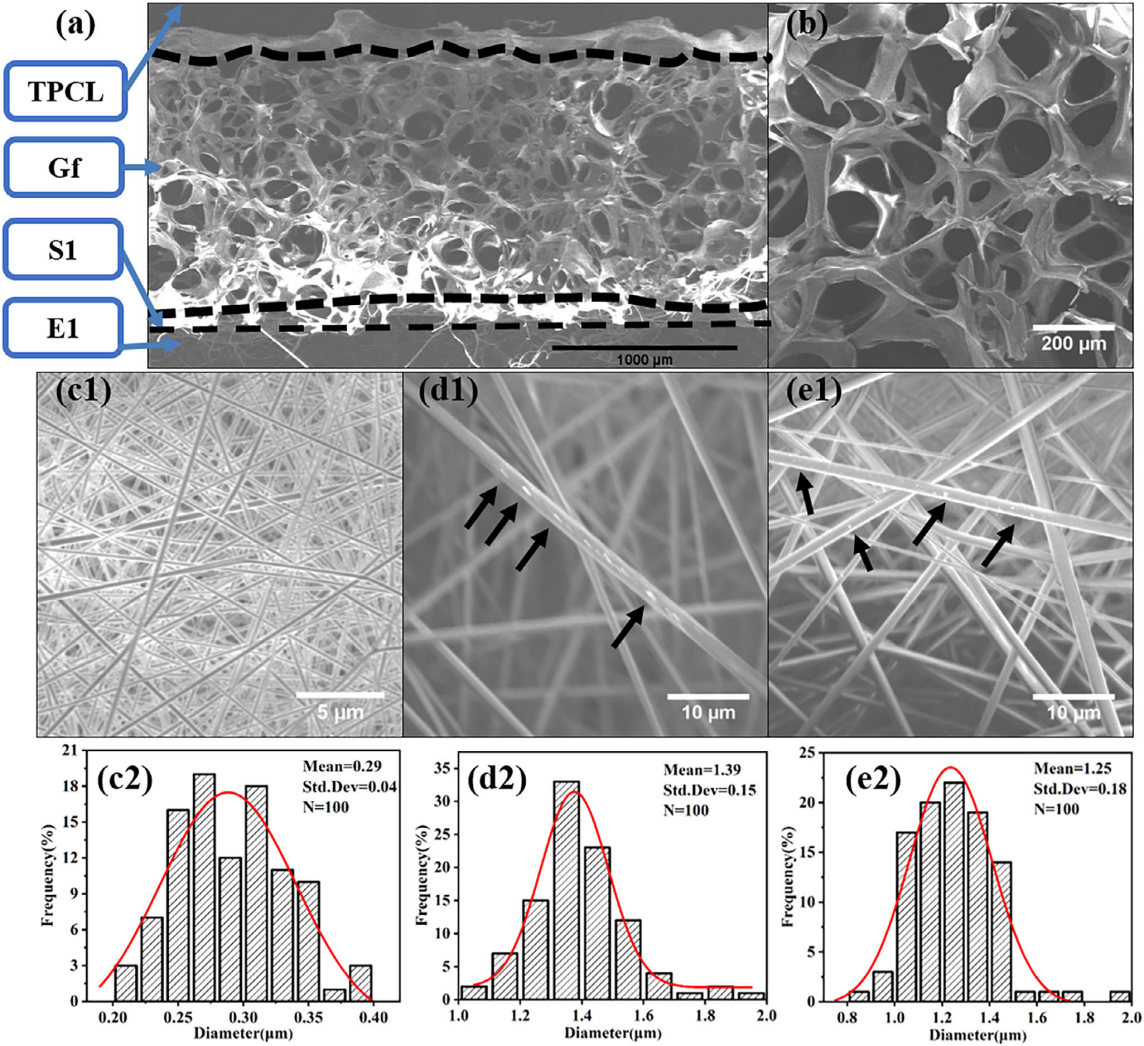
(a) SEM micrograph of the cross‐section of the hierarchical composite. (b) Surface morphology of Gf depicted in an SEM image. (c1) SEM image of TPCL. (c2) Fiber diameter distribution curves of TPCL. (d1) SEM image of S1. (d2) Fiber diameter distribution curves of S1. (e1) SEM image of E1. (e2) Fiber diameter distribution curves of E1. (Black arrows indicate the drug‐loaded regions).

The cross‐sectional SEM image of Gf‐CR demonstrated an obvious four‐layer structure as shown in Figure 1a. The top layer consisted of PCL (TPCL), which acted as a barrier tightly bonded to the GF layer (Figure 1b) through electrostatic adsorption [30]. The TPCL fibers exhibited a uniform morphology (Figure 1c1) with an average diameter of 0.29 ± 0.04 µm (Figure 1c2). The middle layer, a GF hemostatic sponge, displayed a thickness of 1.25 mm and contained large interconnected pores with an average size of 121.4 ± 31.0 µm (Figure 1b). The bottom layers comprised antimicrobial S100/Rif and anti‐inflammatory E100/Cur fibrous mats. Smooth, bead‐free fibers of S100/Rif and E100/Cur (Figure 1d1–e1) exhibited visible drug inclusions within the fibers, as indicated by the black arrows. The average fiber diameters were 1.39 ± 0.15 µm for S100/Rif and 1.25 ± 0.18 µm for E100/Cur (Figure 1d2–e2). A seamlessly integrated hierarchical multilayer composite with varying GF thicknesses could be fabricated, as shown in Figure S1. The resulting three‐dimensional multilayer composite exhibited a yellowish hue at the bottom, attributed to the encapsulation of curcumin within the anti‐inflammatory E100/Cur layer.

### 2.2 Chemical Characterization

**FIGURE 2:**
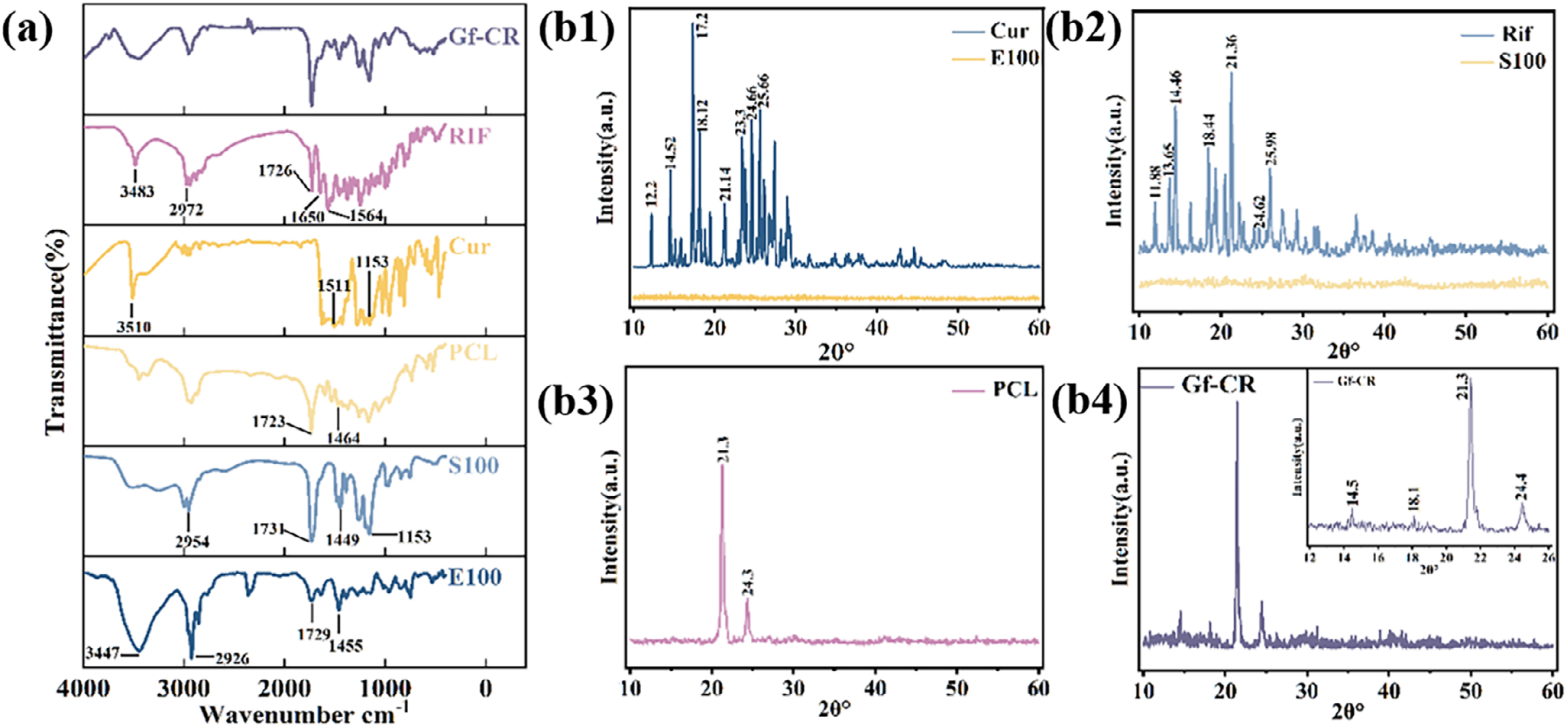
(a) FTIR spectrum of the hierarchical composite. (b1) XRD pattern of Cur and E100. (b2) XRD pattern of Rif and S100. (b3) XRD pattern of PCL. (b4) XRD pattern of Gf‐CR composite.

FTIR analysis was employed to elucidate the chemical bonding patterns between various components of the hierarchical composite. As shown in Figure 2a, E100 exhibited characteristic absorption peaks at 1455, 1729, 2926, and 3447 cm^−1^ corresponded to N─CH_3_ stretching, C─H stretching, C═H stretching, and H─C─H bending, respectively [31]. S100 had characteristic absorption peaks at 1153, 1449, 1731, and 2954 cm^−1^, associated with C‐O esters, esterified carboxylic group, C═O esters, and O─H bonds, respectively [32]. The absorption peaks at 1464 and 1723 cm^−1^ in the PCL spectra correspond to ─CH_2_ and C═O bonds [33]. For Cur, the characteristic absorption peaks at 1153, 1511, and 3510 cm^−1^ represented the stretching vibrations of aromatic C─OH groups, aromatic C─C groups, and phenolic O─H groups, respectively [34, 35]. Rif presented typical peaks at 1556, 1650, 1726, 2972, and 3483 cm^−1^ due to C═C─, ─C═O, C═O, ─OH, and ─NH bonds, respectively [36]. The GF‐CR composite features a four‐layer hierarchical structure composed of PCL, E100, S100, Rifampicin (Rif), Curcumin (Cur), and Gelform. FTIR analysis confirmed the presence of PCL, E100, S100, and Rif in the composite, as all characteristic absorption peaks for these components were observed. This indicates successful drug loading and suggests that the components remained stable during electrospinning.

Figure 2b1‐4 displays the X‐ray spectra in the 2θ range of 10–60° for various pure components and the dual‐drug‐loaded hierarchical composite. As shown in Figure 2b1‐2, E100 and S100 had no distinct characteristic peaks, indicating they were amorphous, while Cur and Rif had crystalline structures. Cur exhibited narrow and sharp diffraction peaks at 2θ = 12.2°, 14.52°, 17.2°, 18.12°, 21.14°, 23.26°, 24.66°, and 25.66° (Figure 2b1) [37]. Similarly, many diffraction peaks were present in Rif (Figure 2b2). As shown in Figure 2b3, PCL also demonstrated crystalline properties with a sharp and well‐defined diffraction peak at 2θ = 21.3° and a peak of relatively low intensity at 2θ = 24.3° [38]. Figure 2b4 shows that the Gf‐CR composite had a higher intensity diffraction peak at 2θ = 21.3°, caused by the superposition of the diffraction peaks of Cur, Rif, and PCL. Additionally, XRD patterns of the GF‐CR composite showed characteristic diffraction peaks near 2θ = 14.5°, 18.1°, and 24.4°, which corresponded to Cur, Rif, and PCL, respectively. These results confirm the successful incorporation of the drugs and indicate that the crystalline structure of the components was preserved during the fabrication process.

### 2.3 Water Contact Angle

**FIGURE 3:**
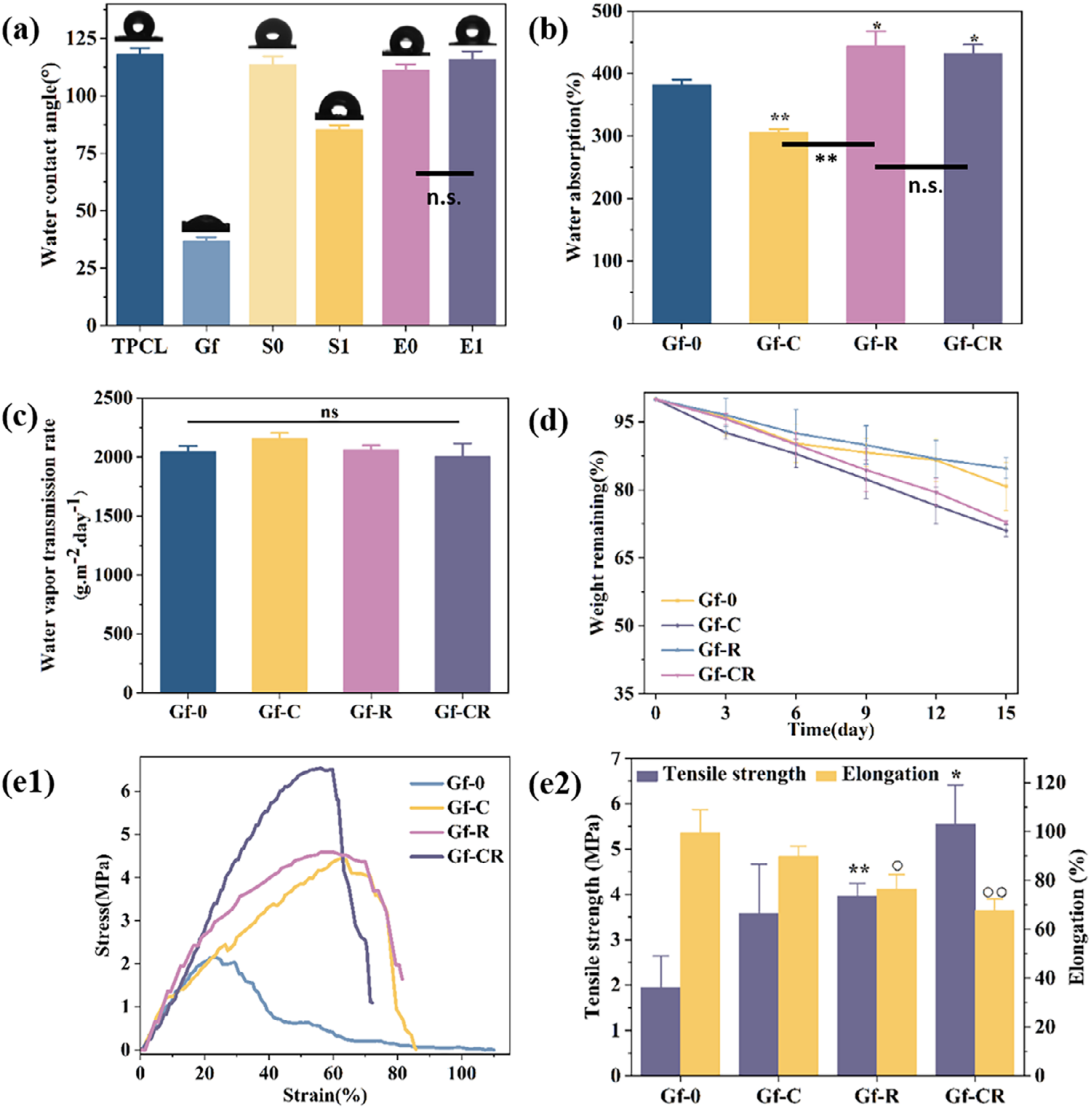
(a) Water contact angles for TPCL, Gf, S0, S1, E0, and E1. (b) Water absorption and (c) water vapor transmission rate for Gf‐0, Gf‐C, Gf‐R, and Gf‐CR. (d) Degradation: % weight remaining of Gf‐0, Gf‐C, Gf‐R, and Gf‐CR. (e1) Representative stress–strain curves for Gf‐0, Gf‐C, Gf‐R, and Gf‐CR. (e2) Tensile strength and elongation values of Gf‐0, Gf‐C, Gf‐R, and Gf‐CR. (°: Compared to Gf‐0 group, *: Compared to Gf‐0 group,* *p* < 0.05, ** *p* < 0.01, *** *p* < 0.001, ° *p* < 0.05, °° *p* < 0.01.).

As shown in Figure 3a, the TPCL layer exhibited a water contact angle of 118.1 ± 2.8°, confirming its hydrophobic nature. This layer functions as a barrier analogous to the epidermis of human skin. The Gf layer demonstrated hydrophilicity, with a water contact angle of 36.8 ± 1.6°. Complete absorption occurred within 4 s, highlighting its strong potential for efficient wound exudate absorption (Figure S2). Upon incorporating the hydrophilic drug Rif into the hydrophobic S0 layer, the hydrophilicity increased, reducing the water contact angle of S1 to 85.3 ± 2.0°. Conversely, E0 and E1 show no significant difference, indicating that the incorporation of Cur has little effect on the hydrophilicity of the composite. This configuration established a hydrophilic gradient from the bottom E1 layer through S1 up to the spongy GF layer in the Gf‐CR composite.

### 2.4 Water Absorption Capacity

An ideal wound dressing should possess a high water absorption capacity to effectively drain and retain wound exudate. As shown in Figure 3b, the water absorption capacity of Gf‐0 (without drug loading) was 382.0 ± 8.1%. Loading only Cur significantly decreased absorption due to the hydrophobic nature of Cur, whereas loading only Rif markedly enhanced absorption, attributed to Rif's hydrophilic properties. For Gf‐CR, which contained both Cur and Rif, the water absorption capacity (431.4 ± 15.1%) was significantly higher than that of Gf‐0. Notably, the absorption capacity of Gf‐CR falls within the optimal range for wound dressings (100–900%) [39]. Figure S3 demonstrates the high absorption capacity of the pure GF layer over time. When horizontal or vertical pressure was applied to the GF layer, the absorbed water was released. This excellent water absorption behavior enables efficient exudate collection, removal of excess fluid, and acceleration of coagulation.

### 2.5 Water Vapor Permeability

Adequate breathability is essential for wound dressings, as it maintains a moist yet ventilated environment conducive to healing [40]. As shown in Figure 3c, the water vapor transmission rates (WVTRs) of Gf‐0, Gf‐C, Gf‐R, and Gf‐CR were 2045.1 ± 47.8, 2155.1 ± 48.6, 2063.6 ± 34.9, and 2003.3 ± 111.5 g·m^−^^2^·day^−^^1^, respectively. The results indicate no significant differences among the samples, suggesting that drug loading did not notably alter the permeability of the composite dressing. All WVTR values fall within the ideal range for wound dressings (2000–2500 g·m^−^^2^·day^−^^1^) [10], confirming that the hierarchical composite provides an optimal microenvironment for wound healing, preventing exudate accumulation and infection while minimizing dehydration.

### 2.6 Degradation of Hierarchical Composite

The degradability of the composite dressing was assessed as shown in Figure 3d. After 15 days of immersion in pure water, the remaining weight percentages for Gf‐0, Gf‐C, Gf‐R, and Gf‐CR were 80.8 ± 5.4%, 71.0 ± 1.3%, 84.8 ± 2.4%, and 72.9 ± 0.4%, respectively. There was no significant difference among the groups, since the only variable was the loading amount of Cur and Rif, while the other composite components remained the same. Therefore, the small quantities of these drugs did not significantly affect the degradation of the composite. Despite these variations, the slow and controlled degradation observed in all composites is in line with the biodegradation requirements for wound dressings [41]. The slow and controlled degradation observed in all composites is consistent with the biodegradation requirements for wound dressings [42].

### 2.7 Mechanical Properties of Hierarchical Composite

An effective wound dressing must exhibit sufficient mechanical strength to resist rupture during handling and application, as well as flexibility to withstand deformation, abrasion, and mechanical stress from joint movement [43]. As shown in Figure 3e1, the stress–strain curves of Gf‐0, Gf‐C, Gf‐R, and Gf‐CR revealed that Gf‐CR possessed the highest Young's modulus among all composites (Figure S4). The Gf‐CR exhibited a significantly higher tensile strength compared to the unloaded Gf samples (Figure 3e2). Co‐loading Cur and Rif significantly increased tensile strength, although it slightly reduced elongation at break, likely due to drug incorporation within the fiber matrix. The Gf‐CR composite exhibited a tensile strength of 5.5 ± 0.9 MPa and an elongation at break of 67.9 ± 4.5%, values that fall within the optimal range for wound dressings (tensile strength: 5–30 MPa; elongation: 35%–115%) [44]. The results of wet‐state tensile properties of the four‐layer hierarchical composites are presented in Figure S5. As shown, the tensile strength and elongation at break of all samples (Gf‐0, Gf‐C, Gf‐R, and Gf‐CR) decreased significantly under wet conditions compared to their dry‐state values. Furthermore, no significant differences in mechanical performance were observed among the different wet‐state sample groups. Figure S6 indicates the delamination occurred at a drawing force of 2.86 N. This value suggests that the adhesive strength of the composite is sufficient to maintain its layered integrity during the wound‐healing process when applied to superficial skin wounds.

### 2.8 Driving Force Mechanism of Directional Flow

**FIGURE 4:**
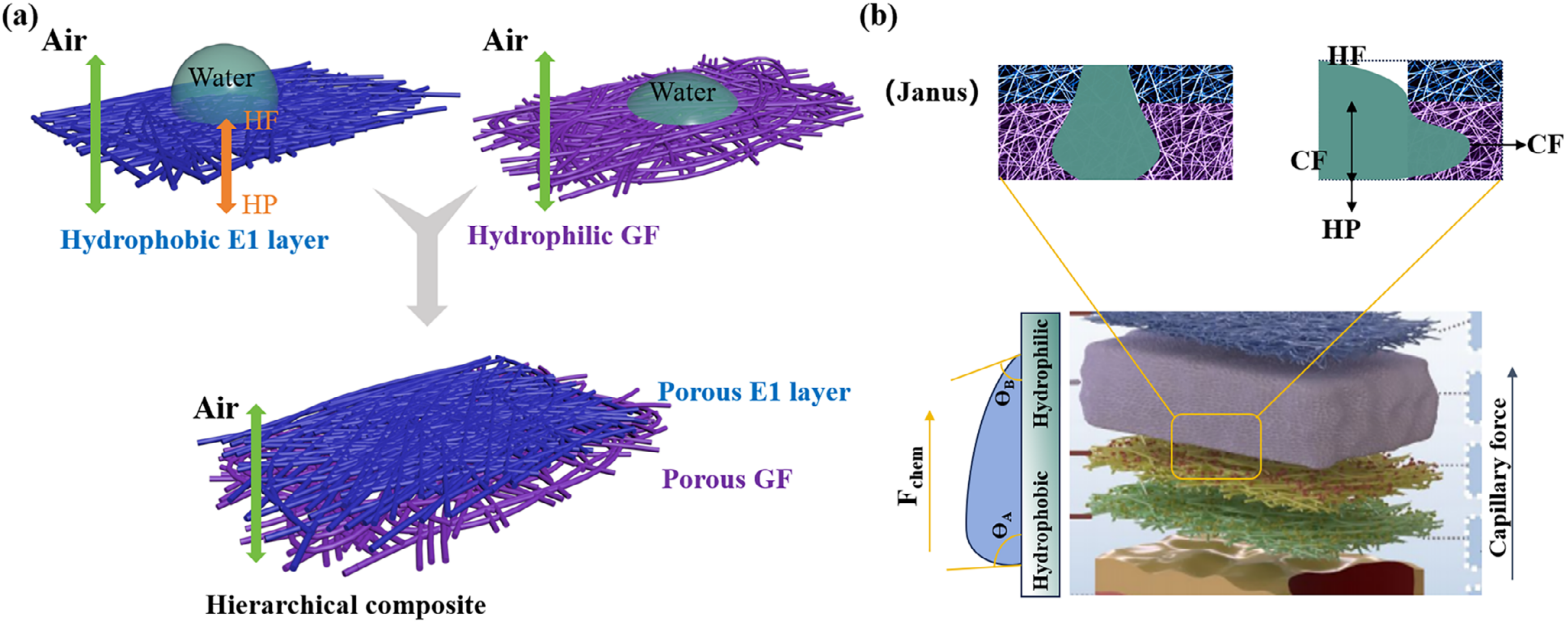
Schematic illustration of the directional flow mechanism in the hierarchical composite. (a) The structure comprises a hydrophobic (E1) layer and gradient hydrophilic S1 and GF layers. (b) A directional driving force facilitates guided liquid transport from the hydrophobic to the hydrophilic region.

The mechanism of directional flow in the hierarchical composite is illustrated in Figure 4. As shown in Figure 4a, the composite comprises a hydrophobic (E1) layer and gradient hydrophilic S1 and GL layers, exhibiting a gradual transition in surface wettability from the E1 layer (113°) to the S1 layer (85°) and finally to the GL layer (35°). Upon initial contact with the hydrophobic E1 layer, liquid penetration is driven by hydrostatic pressure (HP) and resisted by hydrophobic force (HF). Although HF continuously opposes penetration, the accumulating liquid on the surface enhances the effective penetration force. As a result, the liquid is subjected to a net driving force that directs it toward the more hydrophilic region and ultimately into the GF layer. This process arises from the combined effects of the surface energy gradient, hydrostatic pressure, and capillary flow.

As illustrated in Figure 4b, the surface energy and Laplace pressure gradients jointly generate a directional driving force that enables guided liquid transport. The surface energy gradient creates a wettability difference that drives liquid from hydrophobic to hydrophilic regions. The driving force generated by the wettability gradient can be expressed as: [45, 46]

(1) $$F_{chem\;}\approx \;\pi R_{0}\gamma \left(cos\theta_{B}-cos\theta_{A}\right)$$

where θ_*B*_ and θ_*A*_ denote the contact angles of droplets on the hydrophilic and hydrophobic surfaces, respectively; *R*_0_ is the droplet base radius and γ is the liquid surface tension.

The Laplace pressure gradient primarily arises from the asymmetric geometry of the composite. When liquid contacts the porous, hydrophilic G layer, capillary forces (CF) promote spontaneous infiltration. As shown in Figure 2a, the hydrophobic E1 layer is significantly thinner than the hydrophilic S1 and GF layers. Once the penetration depth reaches the layer interface, the liquid contacts the underlying hydrophilic GF layer, which generates an additional pressure gradient that promotes unidirectional flow while inhibiting reverse flow, functioning analogously to a liquid diode [47]. Together with HP, CF enables continuous liquid penetration. The asymmetric structure also induces a curvature gradient, which drives directional motion along the fibers. Droplet motion is initially driven by the contact angle difference between its two ends. As the motion progresses, curvature variations along the liquid–air interface generate a Laplace pressure difference, which subsequently sustains autonomous movement. On hydrophilic surfaces, droplets exhibit a relatively low growth rate but higher velocity, whereas the opposite behavior is observed on hydrophobic surfaces. Studies on asymmetric structures and wettability further indicate that surfaces with a well‐defined wettability gradient promote both faster droplet growth and enhanced droplet mobility [48, 49]. Meanwhile, the interconnected porous network within the fiber layers can be conceptualized as a bundle of microcapillaries, where capillary forces arise spontaneously to drive liquid transport [50, 51]. This effect arises from the higher Laplace pressure generated by the smaller curvature at the fiber apex, forming a pressure gradient that propels liquid in one direction. The driving force associated with Laplace pressure can be expressed as: [52]

(2) $$F_{Laplace}\approx \int_{r_{1}}^{r_{2}}\frac{2r}{{\left(r+R_{0}\right)}^{2}}sin\alpha dr$$

where r represents the local radius of the conical structure; R_0_ is the radius of the droplet; α is the half‐apex angle of the cone; dr denotes the infinitesimal length element along the cone; and r_2_ and r_1_ are the local radii on the opposite sides of the microdroplet.

The hierarchical composite functions as a thin, porous liquid diode exhibiting Janus wettability, with a hydrophobic surface on one side and a hydrophilic surface on the other [53]. This anisotropic wettability facilitates directional liquid transport from the hydrophobic toward the hydrophilic side while effectively preventing reverse flow [54]. The liquid‐diode behavior of the hierarchical composite is demonstrated in Figure S7a,c. When a PBS droplet (pH 7.4) was placed on the hydrophobic E1 layer, it was rapidly absorbed by the underlying S1 and GF layers, fully infiltrating the structure within 25 min. In contrast, a droplet placed on the TPCL layer remained stationary without infiltration (Figure S7b,d). This unidirectional fluid transport is highly beneficial for wound healing, as it enables exudate to move from the wound‐contact layer into the absorbent GF layer while preventing backflow. Moreover, the top hydrophobic TPCL layer helps retain optimal moisture levels, thereby potentially reducing inflammation and minimizing scar formation [55].

### 2.9 In Vitro Release Studies

**FIGURE 5:**
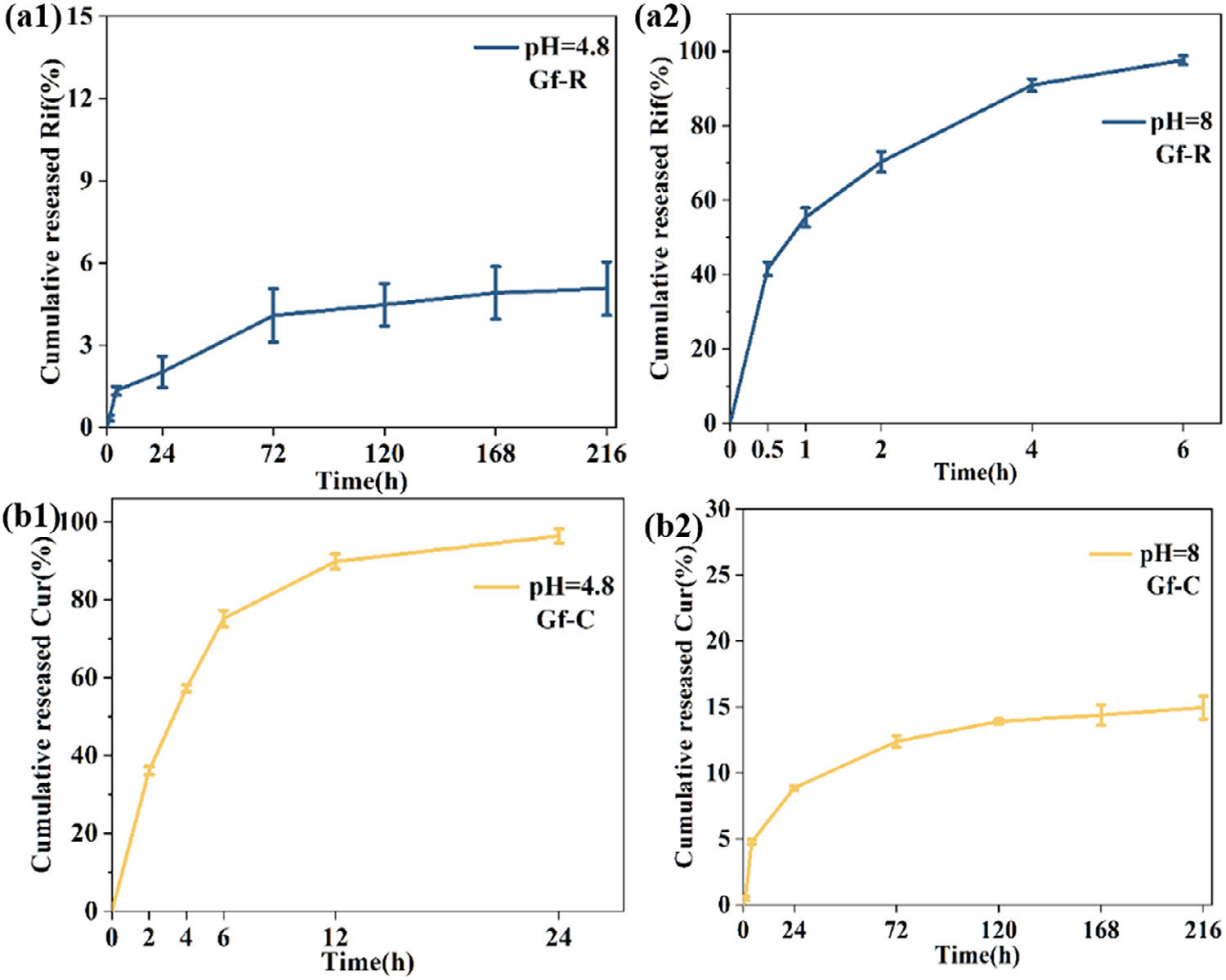
In vitro drug release profiles of Rif from Gf‐R at (a1) pH 4.8 and (a2) pH 8.0, and Cur from Gf‐C at (b1) pH 4.8 and (b2) pH 8.0.

To achieve both antibacterial and anti‐inflammatory effects, the hierarchical composite was loaded with the antibacterial drug rifampicin (Rif) and the anti‐inflammatory drug curcumin (Cur). S100 and E100 served as drug carriers, enabling pH‐responsive release of Rif and Cur, respectively. Figure 5 presents the release profiles of Rif and Cur from the composite in different media. The release of Rif in sodium acetate buffer (pH 4.8) was very slow, with only 5.1 ± 1.0% released over 216 h (Figure 5a1). In contrast, in alkaline PBS (pH 8.0), 97.6 ± 1.2% of Rif was released from Gf‐R within 6 h (Figure 5a2). Similarly, Cur exhibited distinct pH‐responsive release behaviors. As shown in Figure 5b1, in acidic media, Cur release was rapid and substantial, with 96.4 ± 1.8% released from Gf‐R within one day. However, in alkaline media (Figure 5b2), only 15.0 ± 0.8% of Cur was released after nine days. These results demonstrate that the composite achieved effective on‐demand release of both antimicrobial and anti‐inflammatory agents. Such independent drug release behavior can inhibit bacterial infection, alleviate inflammation, and minimize potential side effects associated with uncontrolled drug release. Furthermore, the cumulative release data for Rif and Cur were fitted using the Korsmeyer–Peppas equation (Table 1). The diffusion exponents (n) for Gf‐R and Gf‐C were both below 0.45 in all media, indicating that the drug release followed a Fickian diffusion mechanism [56].

**TABLE 1:** Regression coefficients (R^2^) and diffusion exponents (n) for Rif and Cur released from the hierarchical composite, calculated using the Korsmeyer–Peppas equation.

The pH value of release media	Composite skin scaffolds	R^2^	n
pH = 4.8	Gf‐R	0.90	0.43
	Gf‐C	0.87	0.36
pH = 8	Gf‐R	0.98	0.33
	Gf‐C	0.92	0.36

### 2.10 In Vitro Whole Blood Clotting Tests

**FIGURE 6:**
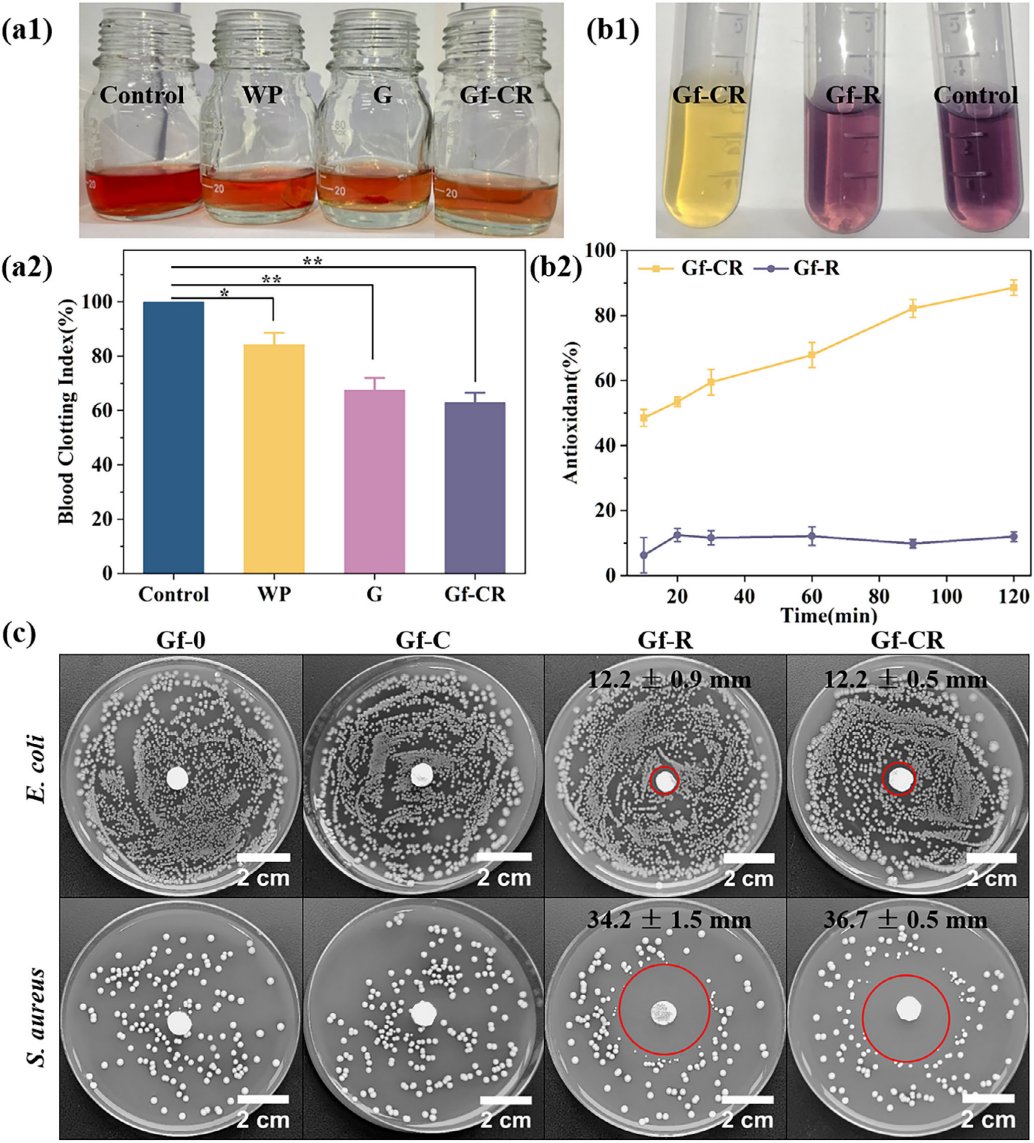
(a1) Optical photographs and (a2) quantitative results of in vitro whole blood clotting test for WP, G, and Gf‐CR. (b1) Optical photographs and (b2) quantitative results of DPPH radical scavenging assay for Gf‐R and Gf‐CR. (c) Antibacterial activity of Gf‐0, Gf‐C, Gf‐R, and Gf‐CR against *E. coli* and *S. aureus*. (Inhibition Zone Diameters in mm. * *p* < 0.05, ** *p* < 0.01, *** *p* < 0.001.).

In vitro whole blood clotting tests were performed to evaluate the hemostatic capability of the hierarchical composite upon contact with blood. Optical images (Figure 6a1) showed markedly lighter solutions in the wound plaster (WP), gauze (G), and Gf‐CR groups compared with the control. Among them, the Gf‐CR group exhibited the lightest color, indicating the most effective blood absorption and clot formation [57]. Quantitative analysis (Figure 6a2) further supported these observations, as the Gf‐CR group demonstrated the lowest blood clotting index (BCI), confirming its superior hemostatic performance. This enhanced coagulation ability is primarily attributed to the gelatin sponge within the hemostatic layer, which facilitates platelet adhesion, aggregation, and clot stabilization [58]. These findings demonstrate the significant hemostatic potential of the Gf‐CR composite for clinical applications.

### 2.11 DPPH radical scavenging assay

The antioxidant activity of the hierarchical composite was evaluated using the DPPH radical scavenging assay. As shown in Figure 6b1, the solution of the Gf‐R group displayed a color similar to that of the control, retaining the characteristic purple–black appearance of DPPH. Correspondingly, Figure 6b2 indicates that the antioxidant activity of Gf‐R remained low throughout the experimental period. In contrast, the DPPH solution treated with Gf‐CR completely faded its color, reflecting strong radical scavenging activity. Notably, the antioxidant capacity of Gf‐CR increased progressively over time, confirming that Cur retained its biological activity after fabrication [59]. After 120 min, Gf‐CR exhibited the highest antioxidant capacity, with an activity of 88.6 ± 2.4%. These results demonstrate that Gf‐CR possesses strong antioxidant activity capable of protecting cells from free radical–induced damage. Consequently, the composite is expected to mitigate oxidative stress [58, 60] and reduce inflammation associated with free radical–mediated cellular injury [61].

### 2.12 Antibacterial Performance

Figure 6c illustrates the antimicrobial efficacy of Gf‐CR composite against *E. coli* and *S. aureus*. The Gf‐0 and Gf‐C samples exhibited no detectable antibacterial activity against either strain, whereas both Gf‐R and Gf‐CR displayed pronounced inhibition zones exceeding 12 mm in diameter, demonstrating strong antimicrobial effects. This activity can be attributed to the presence of Rif in these formulations. Notably, the antibacterial effect against *S. aureus* was significantly greater than that against E. coli, likely due to Rif's enhanced penetration through the *S. aureus* cell wall [62]. In terms of long‐term efficacy, the drug release profiles revealed distinct pH‐dependent behavior (Figure 5). Under acidic conditions (sodium acetate buffer, pH 4.8), Rif release was markedly sustained, with only 5.1 ± 1.0% released after 216 h (Figure 5a1). In contrast, in alkaline PBS (pH 8.0), Gf‐R exhibited rapid release, with 97.6 ± 1.2% of Rif released within just 6 h (Figure 5a2). These results indicate that the Gf‐CR composite can provide prolonged antimicrobial protection in the acidic microenvironment typical of wound inflammation.

### 2.13 Characterization of Cell Bioactivity

**FIGURE 7:**
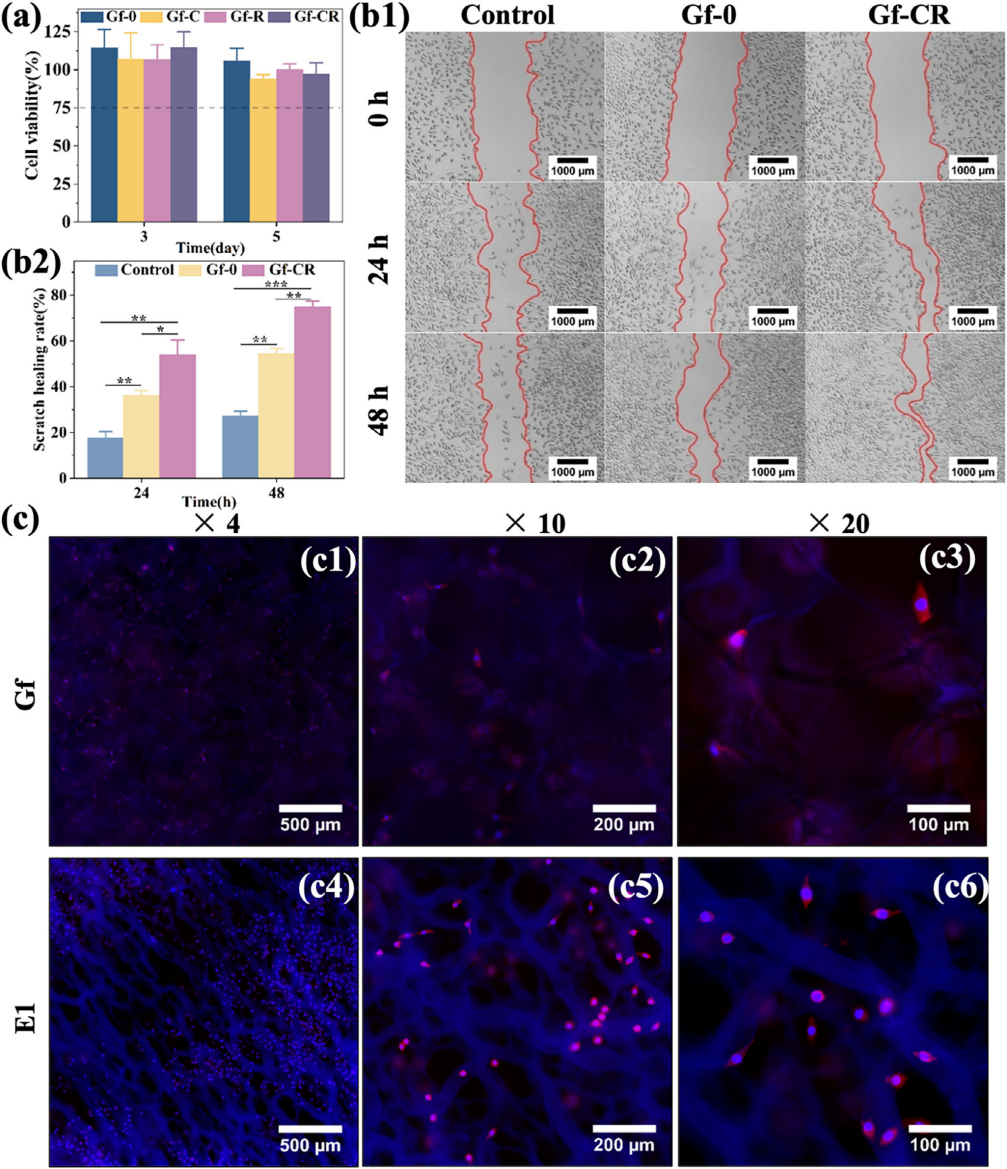
(a) Viability of L929 cells cultured with Gf‐0, Gf‐C, Gf‐R, and Gf‐CR samples. (b1) Representative images showing gap closure of L929 monolayers at 0, 24, and 48 h of incubation. (b2) Quantitative analysis of scratch healing rates in the control, Gf‐0, and Gf‐CR groups. (c) Fluorescence micrographs of L929 cells cultured on the Gf layer (c1–c3) and E1 layer (c4–c6). (*p < 0.05, **p < 0.01, ***p < 0.001).

The results of the CCK‐8 assay (Figure 7a) revealed that cell viability remained above 75% across all groups on days 3 and 5, indicating the absence of significant cytotoxicity toward L929 cells [63]. In particular, the Gf‐CR group exhibited a cell viability of 114.6 ± 10.4% on day 3. Although this value decreased slightly to 97.1 ± 7.5% by day 5, it remained close to 100%, confirming the excellent biocompatibility of the Gf‐CR sample with L929 cells.

The effect of Gf‐CR on L929 cell migration was further assessed using a scratch assay. After 48 h of culture, cells at the wound edges in all groups migrated noticeably toward the scratch center (Figure 7b1 and Figure S8a). As shown in Figure 7b2, the scratch healing rates of the Gf‐CR group reached 54.2 ± 6.3% at 24 h and 75.0 ± 2.5% at 48 h, both significantly higher than those in the control and Gf‐0 groups. When compared with the Gf‐C and Gf‐R groups, which showed healing rates of 63.4 ± 1.2% and 61.4 ± 1.2% after 48 h (Figure S8b), the Gf‐CR group exhibited superior wound closure performance. These results demonstrate that the combined incorporation of Cur and Rif synergistically promotes cell migration. To further investigate cell morphology, L929 cells cultured on the contact layer (E1) and Gf layer were fluorescently stained. As shown in Figure 7c, cells exhibited random yet well‐spread growth along the porous structure of the Gf layer and the fibrous network of the E1 layer, indicating good adhesion and healthy morphology. Thus, these findings confirm that the Gf‐CR composite possesses excellent biocompatibility and effectively supports cell adhesion and migration, underscoring its potential as a promising wound dressing material.

### 2.14 In Vivo Wound Healing

**FIGURE 8:**
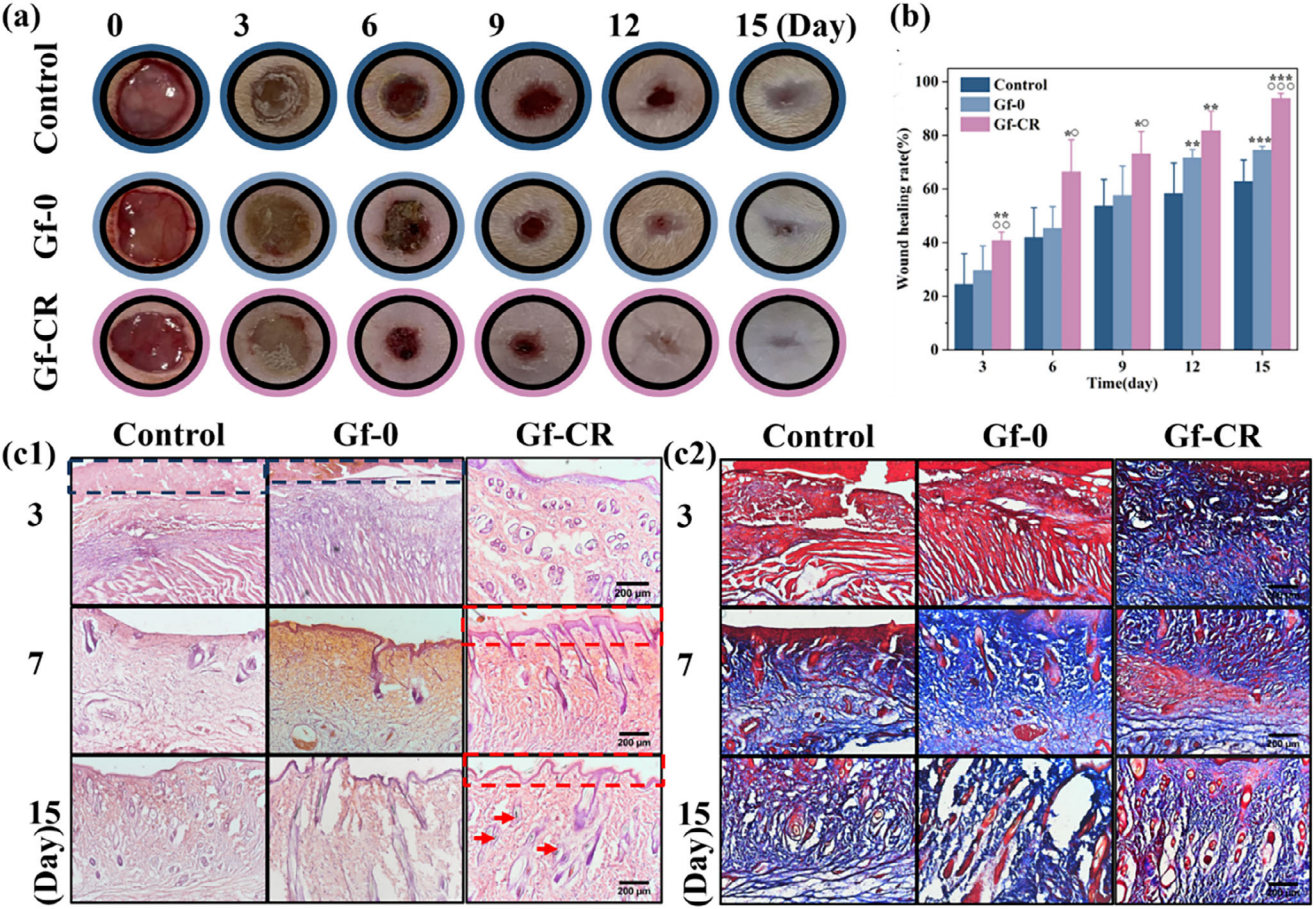
In vivo evaluation of hierarchical composite in promoting full‐thickness wound healing. (a) Representative wound images and (b) quantitative analysis of wound closure (%) in rats treated with Gf‐0 and Gf‐CR at 0, 3, 6, 9, 12, and 15 days post‐implantation. (The diameter of the black circle is 10 mm.) (c1) H&E staining and (c2) Masson's trichrome (MT) staining of skin tissues from the control, Gf‐0, and Gf‐CR groups at different time points after treatment. (* represents comparison with the control group; represents comparison with the Gf‐0 group. *p < 0.05, **p < 0.01, ***p < 0.001.).

The in vivo wound healing performance of the hierarchical composite was evaluated using a full‐thickness skin defect model. Wounds were treated with three groups: no dressing (control), Gf‐0, and Gf‐CR. As shown in Figure 8a, the wound area in all groups progressively decreased over time. However, from day 3 onward (Figure 8b), the Gf‐CR group exhibited markedly accelerated wound closure compared with the control and Gf‐0 groups. By day 15, the wound healing rates reached 93.8 ± 1.9% for Gf‐CR, 74.4 ± 1.5% for Gf‐0, and 62.8 ± 8.0% for the control. Notably, wounds treated with Gf‐CR were almost completely closed, demonstrating a superior healing outcome. Although the gel‐based hierarchical structure of Gf‐0 provided some benefit compared with the control, its efficacy remained lower than that of Gf‐CR. The enhanced therapeutic performance of Gf‐CR can be attributed to its synergistic liquid diode–like behavior and dual‐drug delivery capability. The directional liquid transport, analogous to diode behavior, enabled pH‐responsive release of both drugs, thereby exerting synergistic anti‐inflammatory and antibacterial effects that accelerated wound repair.

To further evaluate tissue regeneration, wound samples were stained with H&E and Masson's trichrome (MT). H&E staining on day 3 revealed substantial scar tissue and dense infiltration of inflammatory cells in the control and Gf‐0 groups (blue box in Figure 8c1). In contrast, the Gf‐CR group displayed markedly reduced inflammation, consistent with the antibacterial and anti‐inflammatory activities of Rif and Cur. From day 7 to day 15, the Gf‐CR group exhibited pronounced re‐epithelialization, characterized by the transition from immature thick epithelium to mature thin epithelium (red box in Figure 8c1), a typical indicator of skin resurfacing through new epithelial proliferation [64]. By day 15, the Gf‐CR–treated wounds showed a well‐formed, uniform neogenic epidermis closely resembling normal skin, with visible regeneration of skin appendages such as hair follicles and sebaceous glands. Moreover, MT staining (Figure 8c2) revealed that by day 15, the Gf‐CR group displayed more uniformly distributed and densely packed collagen fibers in the dermal layer, organized in a basket‐weave pattern, indicative of mature collagen remodeling [65]. Collectively, these findings demonstrate that Gf‐CR significantly enhances wound closure, tissue regeneration, and collagen maturation, confirming its superior therapeutic efficacy in promoting skin wound healing.

## 3 Conclusions

In summary, a novel hierarchical composite was successfully developed to accelerate wound healing. The composite features a four‐layer architecture that mimics liquid‐diode behavior, enabling directional liquid transport from hydrophobic to hydrophilic layers and triggering pH‐responsive drug release for anti‐inflammatory and antibacterial effects. This gradient design ensures excellent porosity, water absorption, breathability, and mechanical robustness, while simultaneously minimizing the risk of maceration and maintaining a favorable moist environment for wound repair. Both in vitro and in vivo evaluations demonstrated the composite's exceptional antioxidant and hemostatic activities, along with its ability to significantly reduce infection and inflammation, thereby promoting rapid wound closure and tissue regeneration. Collectively, these results highlight the multifunctional therapeutic potential of this hierarchical composite and underscore its promise as an advanced material for clinical wound management.

## 4 Experimental Section

### 4.1 Materials

Polycaprolactone (PCL, Mn≈80,000), rifampicin (Rif), and glacial acetic acid (GAA) were purchased from Sigma–Aldrich (St. Louis, MO, USA). Eudragit S100 (S100) and Eudragit E100 (E100) were generously donated by Evonik Industries AG (Essen, Germany). Curcumin (Cur) was purchased from Sinopharm Chemical Reagent Co., Ltd. (China). Gelfoam (Gf), 2,2‐diphenyl‐1‐picrylhydrazyl (DPPH), and dimethylformamide (DMF) were obtained from Shanghai Macklin Biochemical Co., Ltd. (China). Phosphate‐buffered saline (PBS, pH = 8), sodium acetate buffer (pH = 4.8), and calcium chloride (CaCl_2_) solution were purchased from Wanzai County Pinggen Teaching Instruments Sales Department (Yichun City, Jiangxi Province, China).

Sterile individual New Zealand rabbit blood (BC‐AC‐NZR01) was purchased from Nanjing SenBeiJia Biological Technology Co., Ltd. (China). Modified Eagle's Medium (MEM) was obtained from Zhejiang Geno Biomedical Technology Co., Ltd. (China). Heat‐inactivated fetal bovine serum (FBS) was purchased from Zhejiang Tianhang Biotechnology Co., Ltd. (China). Penicillin‐streptomycin mixtures, L‐alanyl‐L‐glutamine, and sodium pyruvate were purchased from Shanghai Zhong Qiao Xin Zhou Biotechnology Co., Ltd. (China). Alexa Fluor 546 phalloidin was obtained from Yeasen Biotechnology Co., Ltd. (Shanghai, China). 4',6‐diamidino‐2‐phenylindole hydrochloride (DAPI) was provided by Solarbio Technology Co., Ltd. (Beijing, China). All materials were used as received without further purification treatment.

### 4.2 Solution Preparation

A PCL/GAA solution was prepared by dissolving PCL (18% w/w) in GAA. S100 (20% w/w) and E100 (15% w/w) were dissolved in DMF to prepare S100/DMF and E100/DMF solutions, respectively. For antimicrobial and anti‐inflammatory purposes, S100/Rif and E100/Cur solutions were prepared by adding 3% and 0.5% (relative to the weight of the polymer) of Rif and Cur to the S100/DMF and E100/DMF solutions, respectively. All solutions were mixed homogeneously at room temperature using a magnetic stirrer (VELP ARE, Italy).

### 4.3 Preparation of Hierarchical Composite

The fabrication process of the hierarchical composite structure is illustrated in Scheme 1. The dressings consisted of four layers, arranged from bottom to top: the anti‐inflammatory layer, antimicrobial layer, hemostatic layer, and barrier layer. The top PCL barrier layer (named TPCL) was a hydrophobic dense membrane fabricated by electrospinning PCL/GAA solution. The solution flow rate was controlled at 80–100 µL/h at 6 kV. This layer facilitated moisture retention, preventing dehydration and microbial infiltration. Beneath the barrier layer was the hemostatic layer composed of Gelfoam (Gf), which featured biocompatible large pores and excellent permeability. The thickness of the gelform was adjustable, and 1.25 mm was selected for this study based on previous research [66]. This layer effectively absorbed blood and local exudate from the wound, creating a moist environment conducive to wound healing. The combination of the barrier layer and the hemostatic layer mimicked the properties of the skin's epidermis.

Then, a Rif‐loaded S100 electrospun membrane (referred to as S1) was produced as an antimicrobial layer using the S100/Rif solution. The solution flow rate at this stage was 60 µL/h, with an applied voltage of 6.25 kV. As a control group, a pure S100 membrane, denoted as S0, was also produced. Subsequently, the anti‐inflammatory layer, named E1, was created at the bottom using E100/Cur nanofiber membranes. The electrospinning parameters were set to 60 µL/h and 6.38 kV. The pure E100 membrane served as the control group, referred to as E0. For all layers, the electrospinning time was maintained at 1 h, and the distance between the nozzle and the receiving platform was adjusted within the range of 6–8 cm. The dual‐drug loaded multi‐layer dressings were referred to as Gf‐CR, while the single‐loaded dressings with Cur and Rif were named Gf‐C and Gf‐R, respectively. Multi‐layer dressings without drug loading were denoted as Gf‐0.

### 4.4 Surface and FTIR Characterization

The morphology of the hierarchical composite was examined using a scanning electron microscope (SEM, TESCAN GAIA3, Czech Republic) at an accelerating voltage of 3 kV. The chemical properties of the composite dressings were analyzed within the range of 0∼4000 cm^−1^ using a Fourier transform infrared (FTIR) spectrophotometer (Bruker Corporation, Zürich, Switzerland).

### 4.5 Water Contact Angle

The hydrophilicity of the hierarchical structure was evaluated using a contact angle and interfacial tension analyzer (DAS30, KRUSS, Germany) with a 5 µL water droplet. Contact angle images were analyzed using Image J software (National Institutes of Health, Bethesda, MD, USA).

### 4.6 Water Absorption

Four samples of hierarchical structure (Gf‐0, Gf‐C, Gf‐R, and Gf‐CR), each measuring 10 × 10 mm^2^, were weighed to determine their dry weight (denoted as *W*_1_,). Subsequently, they were placed in separate glass vials containing 5 mL of deionized water and incubated in a thermostat at 37°C. After 30 min of absorption, excess water was removed by blotting with filter paper. Meanwhile, the waster absorption of pure GF was also investigated in this study. The samples were then reweighed to determine their wet weight (denoted as *W*_2_.). The percentage of water absorption was calculated using the following Equation (3): [67]

(3) $$W\left(\%\right)=\frac{W_{2}-W_{1}}{W_{1}}\times 100\%$$

### 4.7 Water Vapor Transmission Rate

Uniform‐sized samples of Gf‐0, Gf‐C, Gf‐R, and Gf‐CR were used to seal the mouths of glass bottles at room temperature. Each bottle was filled with 5 mL of deionized water. The weights of the bottles were measured after 1 day using a high precision electronic balance (ME203, Mettler Toledo, Switzerland). The water vapor transmission rate (WVTR) was calculated using Equation (4): [68]

(4) $$WVTR\left(g\cdot cm^{-2}\cdot day^{-1}\right)=\frac{W_{W}}{A}$$

where *W_W_* is the weight loss of water per day, and A is the area of the mouth of the glass bottle.

### 4.8 Degradation

The degradation of the hierarchical structure was investigated by incubating samples of Gf‐0, Gf‐C, Gf‐R, and Gf‐CR in sealed tubes containing deionized water at 37°C. The initial weights of the samples were recorded. At designated time intervals, the samples were removed from the tubes and dried in an oven. Their weights were then determined, and the percentage of weight remaining was calculated.

### 4.9 Mechanical Properties

The mechanical properties of the hierarchical structure were evaluated using a moving fixture (JSV‐H1000, Japan Instrumentation System Co., Ltd.) and a force gauge (HF‐1, Japan Instrumentation System Co., Ltd.). Prior to testing, samples of Gf‐0, Gf‐C, Gf‐R, and Gf‐CR were cut to dimensions of 20 mm × 20 mm, and their thickness was measured using a digital caliper. Tensile tests were conducted at a constant speed of 50 mm/min at room temperature. The wet‐state tensile properties of the four‐layer hierarchical composites were evaluated according to the following procedure. Samples (Gf‐0, Gf‐C, Gf‐R, and Gf‐CR) were cut into 10 mm × 10 mm sections. To ensure complete hydration, all samples were placed in a sealed tube containing deionized water and incubated at 37°C for 30 min to reach absorption equilibrium. After incubation, surface moisture was gently removed using filter paper to prevent interference with the mechanical test. Tensile testing was performed using a mobile fixture (JSV‐H1000, JISV Instrument Systems Co., Ltd.) and a force gauge (HF‐1, JISV Instrument Systems Co., Ltd.). Both ends of each hydrated sample were fixed to the fixture, and the test was conducted at room temperature at a crosshead speed of 15 mm/min. The delamination resistance of the Gf‐CR composite was evaluated as follows. Samples were cut into 10 mm × 10 mm sections, and their top and bottom layers were fixed onto a mobile fixture (JSV‐H1000, JIS Instrument Systems Co., Ltd.). A tensile test was then performed at room temperature using a force gauge (HF‐1, JIS Instrument Systems Co., Ltd.) at a constant speed of 15 mm/min.

### 4.10 Release Analyses of Rif and Cur

The release of Rif and Cur from the hierarchical structure was evaluated in sodium acetate buffer (pH 4.8) and PBS (pH 8) at 37°C, simulating the environments of wound inflammation and bacterial infection, respectively [11]. Since Rif and Cur exhibit maximum absorption at 340 and 425 nm, respectively, potential interference between their concentrations in the dual drug‐loaded multilayer dressing was considered. Therefore, single‐loaded multilayer dressings Gf‐C and Gf‐R were also analyzed to determine their individual drug release profiles. Samples were cut to dimensions of 10 mm × 10 mm and placed separately in 5 mL of the respective release medium. At specified time intervals, 3 mL of supernatant was collected for UV detection using a UV–vis spectrophotometer (UV‐6100, Metash Instruments Corporation, Shanghai, China) at the wavelengths corresponding to the drugs. An equal volume of fresh medium was then replaced. To determine the kinetic mechanism of drug release for Rif and Cur, the obtained release data were fitted to the Korsmeyer‐Peppas equation (Equation (5)): [69]

(5) $$\frac{M_{t}}{M_{\infty }}=kt^{n}$$

where $\frac{M_{t}}{M_{\infty }}$ represents the fraction of drug release at time t, k is a characteristic constant, and n is the release exponent that characterizes the release mechanism.

### 4.11 In Vitro Whole Blood Clotting Test

The hierarchical composite was cut to a size of 1 cm × 1 cm and placed in a glass vial. A separate glass vial without the sample served as a control. Pre‐incubation was conducted at 37°C for 5 min. Then, 100 µL of fresh anticoagulated rabbit blood (Nanjing Senbeijia Biotechnology Co., Ltd, China) was applied to the surface of the sample, followed by the addition of 20 µL of 0.2 M CaCl2 solution. After incubating for an additional 5 min at 37°C, 25 mL of distilled water was added to the glass vial. The vial was then placed on a thermostatic oscillator and oscillated at 100 rpm for 5 min. Finally, the absorbance of the samples was measured at λ = 545 nm using a UV–vis spectrophotometer (UV‐6100, Metash Instruments Corporation, Shanghai, China). The blood‐clotting index (BCI) was calculated using the following equation: [57]

(6) $$BCI\;\left(\%\right)=\frac{A_{1}-A_{0}}{A_{0}}\times 100\%$$

where *A*_0_ and *A*_1_ are the absorbance of the control and experimental groups, respectively.

### 4.12 DPPH Radical Scavenging Assay

A 25 mg sample of the hierarchical composite was immersed in a test tube containing 100 µM methanol DPPH solution and treated for 1 h at 37°C. Pure methanol DPPH solution served as a blank control. Subsequently, all tubes were placed at room temperature, and the absorbance of the solution was measured at *λ* = 517 nm every 20 min using a UV–vis spectrophotometer. The antioxidant activity was calculated using the following formula: [59]

(7) $$\mathrm{Antioxidant}\;\mathrm{activity}\;\left(\%\right)=\frac{A_{C}-A_{S}}{A_{C}}\times 100\%$$

where *A_C_* and *A_S_* represent the absorbance of the control and experimental groups, respectively.

### 4.13 Antibacterial Performance Evaluation

The antibacterial activity of the hierarchical composite against Staphylococcus aureus (*S. aureus*, ATCC 6538) and Escherichia coli (*E. coli*, ATCC 29522) was evaluated using the disc diffusion assay. Samples Gf‐0, Gf‐C, Gf‐R, and Gf‐CR were pre‐cut into 10 mm diameter discs and exposed to ultraviolet light for 30 min. Each disc was fully exposed to ensure sterilization. Next, 200 µL of bacterial culture medium (2 × 10^6^ CFU/mL) containing either *S. aureus* or *E. coli* was evenly spread onto separate agar plates. The prepared multilayer dressings were gently placed on the agar surface and incubated at 37°C for 24 h. After incubation, images of the discs were captured using a camera, and the diameter of the inhibition zone around each disc was measured.

### 4.14 L929 Cell Culture

A mouse fibroblast cell line (L929) was used in this study. The cells were cultured in complete medium consisting of 87% MEM, 10% FBS, 1% penicillin‐streptomycin mixtures, 1% L‐alanyl‐L‐glutamine, and 1% sodium pyruvate under conditions of 37°C and 5% CO_2_.

### 4.15 CCK‐8 Cell Viability Test

For the CCK‐8 cell viability test, L929 cells were seeded in 96‐well plates. Samples of Gf‐0 and Gf‐CR were pre‐cut to a size of 3 mm × 3 mm and sterilized by exposure to UV light for 24 h. On the first day, 100 µL of L929 cell suspension at a density of 1 × 10^4^ cells/ml was added to the experimental and control wells. Additionally, 100 µL of complete medium was added to the blank wells. The samples were then added to the experimental wells. After incubating for 3 and 5 days, 10 µL of CCK‐8 reagent was added to each well, and the plates were incubated for an additional 4 h. The absorbance values were measured at 450 nm using a Microplate reader (Nivo, PerkinElmer, USA). The relative cell viability (%) was calculated using the following equation:

(8) $$Cell\;viability\;\left(\%\right)=\frac{{\left[A\right]}_{test}-{\left[A\right]}_{blank}}{{\left[A\right]}_{control}-{\left[A\right]}_{blank}}\times 100\%$$

where [*A*]_*test*_, [*A*]_*control*_, and [*A*]_*blank*_ represent the absorbance values of the experimental, control, and blank wells, respectively.

### 4.16 Cell Migration

Samples of Gf‐0 and Gf‐CR were pre‐cut to dimensions of 10 mm × 10 mm and sterilized by exposure to UV light for 24 h. When L929 cells reached 80% confluence, they were passaged and seeded into 6‐well plates at a density of 3 × 10^5^ cells/well. Once a monolayer of cells was formed, a sterile 200 µL pipette tip was used to create uniform scratches on the cell surface. Subsequently, the pre‐cut samples were placed into the 6‐well plates for co‐culture with L929 cells. At different time intervals, images of the scratch area were captured using an inverted fluorescent microscope (Nikon, Eclipse Ti, Japan). Cell migration rate was calculated using the following equation (Equation (9)): [39]

(9) $$Scratch\;healing\;rate\;\left(\%\right)=\frac{S_{0}-S_{t}}{S_{0}}\times 100\%$$

where *S*_0_ and *S_t_* represent the original scratch area and the scratch area after t hours of incubation, respectively.

### 4.17 Cell Morphology Study

Samples were pre‐cut to dimensions of 10 mm × 10 mm and sterilized by exposure to UV light for 24 h. These samples were then placed in 6‐well plates, and L929 cells were inoculated onto the samples at a density of 1 × 10^5^ cells/cm^2^. After 3 days of culture, the cytoskeleton and nucleus of L929 cells were stained using Phalloidin and DAPI, respectively, to visualize actin filaments and cell nuclei. Fluorescent staining was observed using an inverted fluorescent microscope (Nikon, Eclipse Ti, Japan).

### 4.18 In Vivo Wound Healing

In vivo wound healing experiments were performed using a full‐thickness skin defect model in Sprague–Dawley rats following established protocols [11]. Samples of Gf‐0 and Gf‐CR were pre‐cut into discs (1.25 mm thickness, 10 mm diameter) and sterilized under ultraviolet light overnight. The study was approved by the Biomedical Ethics Committee of Hebei University of Technology (HEBUTACUC2023049) and conducted in accordance with institutional ethical standards and the National Institutes of Health guidelines for the care and use of laboratory animals.

Male Sprague Dawley rats weighing 280–300 g and aged 6–8 weeks were used for the in vivo experiments. Each rat was housed individually at room temperature (relative humidity: 50–60%) with free access to commercial laboratory chow and water until sacrifice by ketamine/xylazine overdose. The animals were divided into three groups: control, Gf‐0, and Gf‐CR groups (*n* = 4). After anesthetizing the rats, their backs were shaved and sterilized. A 10 mm diameter wound was created using sterile ophthalmic scissors, and the wounds were immediately covered with the prepared composite dressings. The control group did not receive any treatment. Photographs of the wounds were taken at 0, 3, 6, 9, 12, and 15 days post‐wounding to monitor wound healing progress. The wound healing rate was calculated using the following formula:

(10) $$Wound\;healing\;rate\;\left(\%\right)=\frac{W_{0}-W_{n}}{W_{0}}\times 100\%$$

where *W*_0_ is the wound exposure area at day 0, *W_n_* is the wound exposure area at day n (*n* = 3, 6, 9, 12, and 15).

After 3, 7, and 15 days, the rats were euthanized, and the skin tissue encompassing the wound area and surrounding skin was promptly excised and fixed in 4% paraformaldehyde. The fixed tissues were then embedded in a freezing embedding agent, and 5 µm thick sections were prepared using a cryostat microtome (Leica CM 1850; Leica Microsystems, Seoul, Korea). For histological analysis, tissue sections were stained with hematoxylin and eosin (H&E) and Masson's trichrome (MT). Stained sections were examined and photographed using an inverted fluorescent microscope (Nikon, Eclipse Ti, Japan).

### 4.19 Statistical Analysis

All experiments were conducted in triplicate, and data were expressed as mean ± standard deviation (SD). Statistical differences between experimental groups were analyzed using one‐way analysis of variance (ANOVA) performed with Origin software. Significance levels were denoted as follows: *p* < 0.05 (*), *p* < 0.01 (**), *p* < 0.001 (***).

## Author Contributions

**Baolin Wang**: Writing – review & editing, Writing – original draft, Methodology, Funding acquisition, Conceptualization. **Li‐fang Zhu**: Investigation, Methodology, Revising, and Editing. **Yuna Lang**: Investigation, Methodology, Writing – original draft, and Data curation. **Siyi Zhang**: Investigation, and Methodology. **Fei Chen**: Investigation, and Methodology. **Ming‐Wei Chang**: Writing – review & editing, Supervision, and Conceptualization.

## Conflicts of Interest

The authors declare no conflicts of interest.

## Supporting information




**Supporting File**: adhm70876‐sup‐0001‐SuppMat.docx.

## Data Availability

The data that supporting the findings of this study are available on request from the corresponding author. The data are not publicly available due to privacy or ethical restrictions.
