# Supplementary material for: Directional Liquid Transport Enabled pH‐Responsive Hierarchical Composite for Enhanced Wound Healing
Source: Adv Healthc Mater. 2026 Feb 3;15(15):e05497. doi: 10.1002/adhm.202505497 (PMC13088756; doi:10.1002/adhm.202505497)
Supplement: Supplementary file 1 — Supporting File: adhm70876‐sup‐0001‐SuppMat.docx. [file ADHM-15-0-s001.docx]

**Supplementary Material**

**Directional Liquid Transport Enabled pH-Responsive Hierarchical Composite for Enhanced Wound Healing**

Baolin Wang ^a,c,d,*^, Li-Fang Zhu ^b^, Yuna Lang ^a,c,d^ , Siyi Zhang ^a,c,d^ , Fei Chen ^a,c,d^, Ming-Wei Chang ^e,*^

^a^ State Key Laboratory of Reliability and Intelligence of Electrical Equipment, Hebei University of Technology, Tianjin, 300401, China

^b^ College of Tourism and Leisure Management, Fujian Business University, Fuzhou, Fujian, 350001, China

^c^ Tianjin Key Laboratory of Bio-electromagnetic and Neural engineering, Hebei University of Technology, Tianjin, 300132, China

^d^ Hebei Key Laboratory of Bioelectromagnetics and Neuroengineering, School of Health Sciences and Biomedical Engineering, Hebei University of Technology, Tianjin, 300132, China

^e^ Nanotechnology and Integrated Bioengineering Centre, University of Ulster, Belfast BT15 1AP, UK


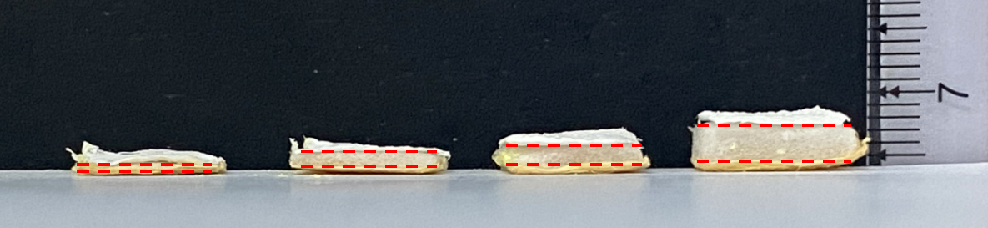


**Fig. S1.** Hierarchical composites with adjustable thicknesses. From left to right, the thicknesses of the Gf layer are 0.5 mm, 1.25 mm, 2.5 mm, and 3.75 mm.


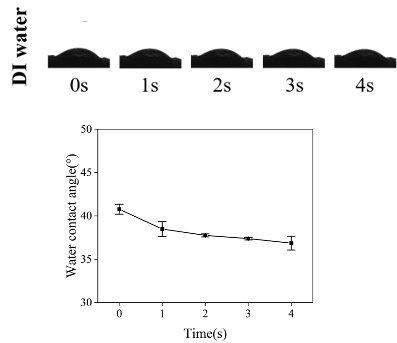


**Fig. S2.** Time-dependent contact angle of the Gf layer.


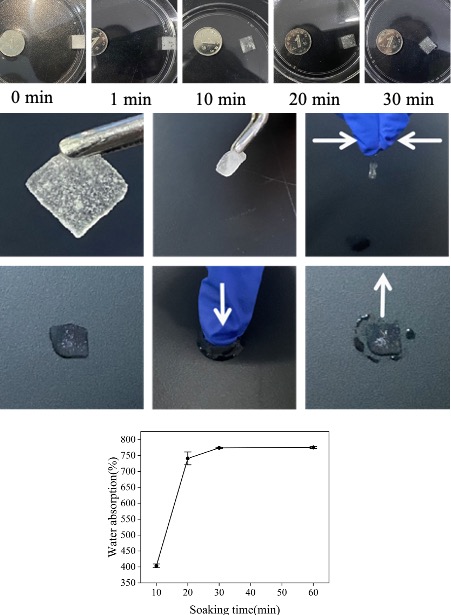


**Fig. S3.** Water absorption of the Gf layer at different time points. After 60 minutes of absorption, pressure was applied either horizontally or vertically to the Gf layer.


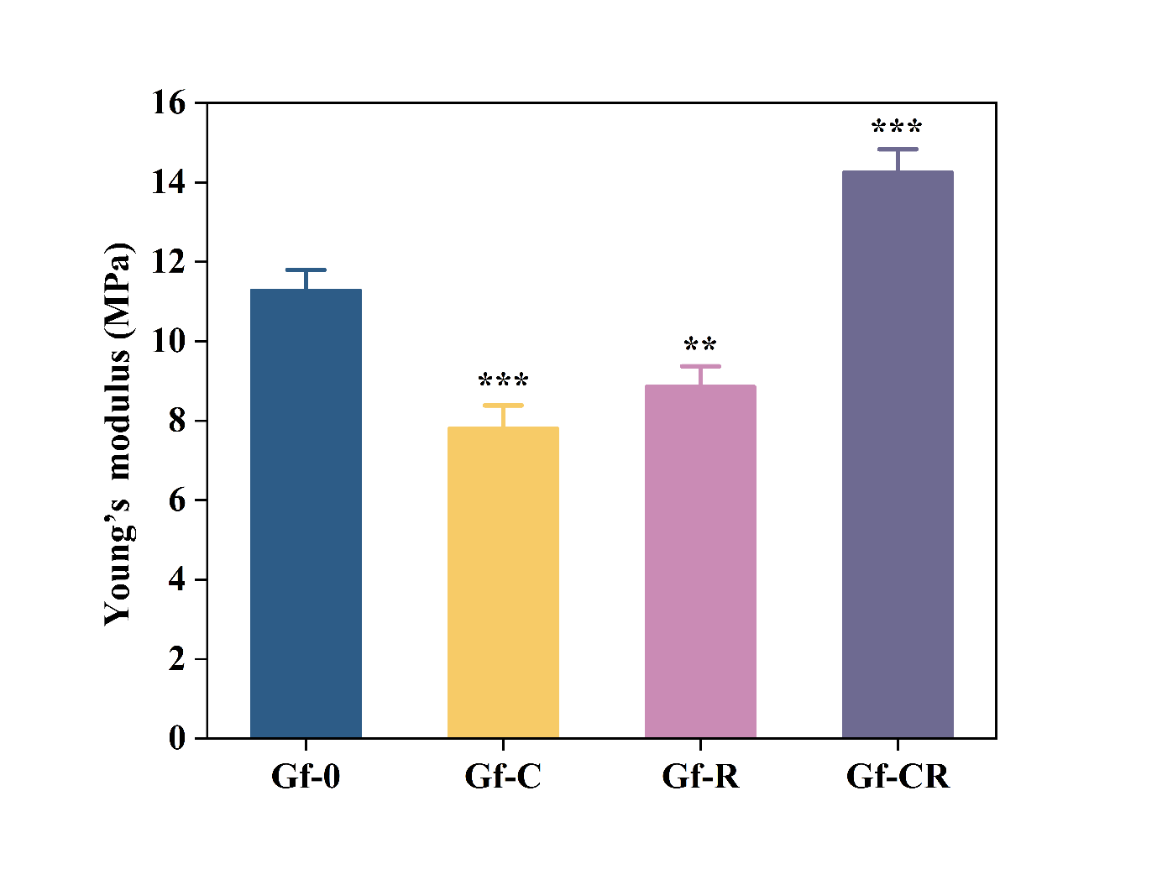


**Fig. S4.** Young’s modulus of Gf-0, Gf-C, Gf-R, and Gf-CR. (* Compared with the Gf-0 group; * p < 0.05, ** p < 0.01, *** p < 0.001)


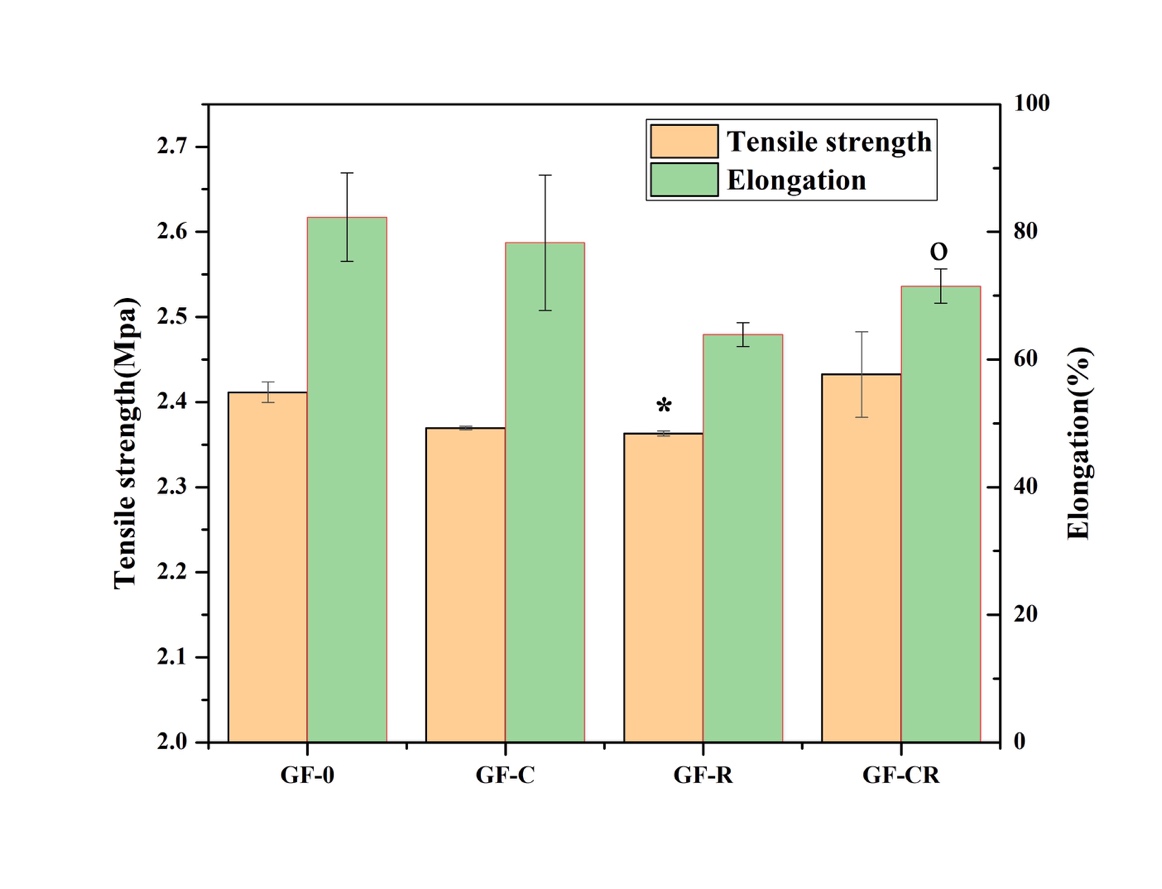


**Fig. S5.** Tensile strength and elongation values of wet-state Gf-0, Gf-C, Gf-R, and Gf-CR. (*: Compared to Gf-0 group, * p < 0.05; ○: Compared to Gf-R group, ○ p < 0.05.)


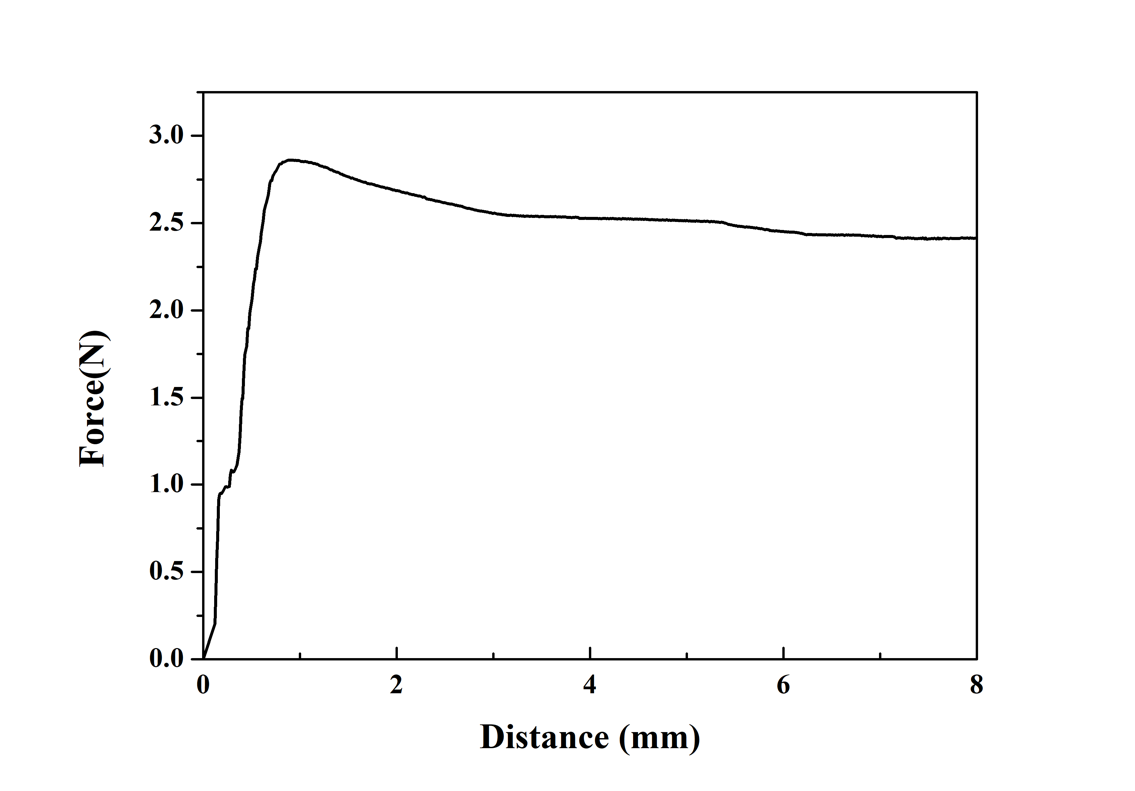


**Fig. S6.** The delamination resistance of the Gf-CR composite.


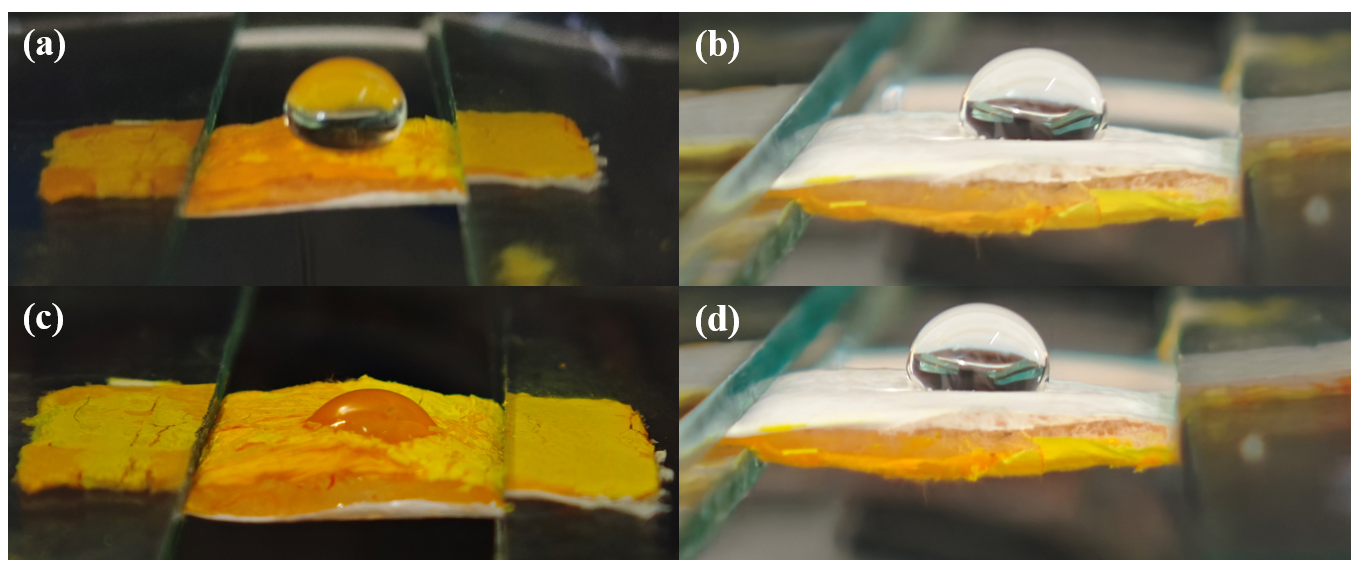


**Fig. S7.** Liquid-diode behavior of the hierarchical composite. (a) Initial deposition of a PBS droplet (pH 7.4) on the anti-inflammatory layer (E1). (b) Initial deposition of a PBS droplet on the barrier (TPCL) layer. (c) Absorption of the PBS droplet on the anti-inflammatory layer after 25 minutes. (d) PBS droplet remaining on the barrier layer after 25 minutes.

(a)
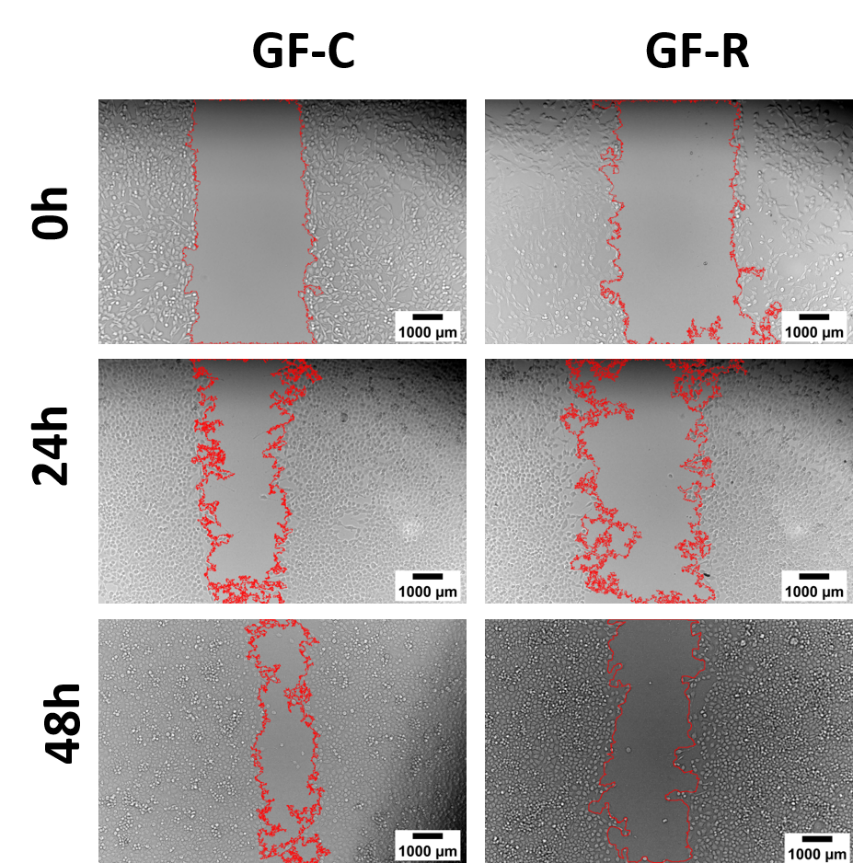


(b)
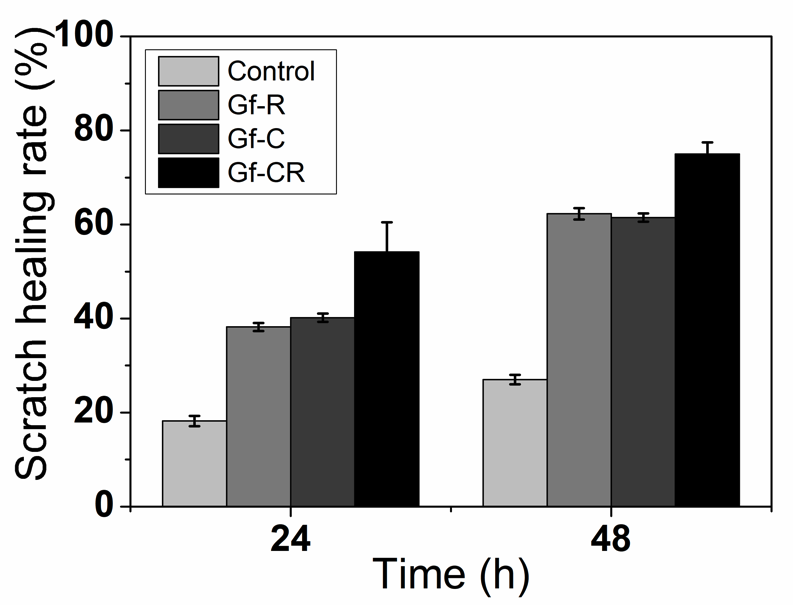


**Fig. S8.** Gap closure of L929 monolayers cultured on Gf-C and Gf-R at 0, 24, and 48 hours of incubation.
